# A novel magnetic resonance imaging scoring system for active and chronic changes in children and adolescents with juvenile idiopathic arthritis of the hip

**DOI:** 10.1007/s00247-022-05502-8

**Published:** 2022-09-23

**Authors:** Laura Tanturri de Horatio, Susan C. Shelmerdine, Paola d’Angelo, Pier Luigi Di Paolo, Silvia Magni-Manzoni, Clara Malattia, Maria Beatrice Damasio, Paolo Tomà, Derk Avenarius, Karen Rosendahl

**Affiliations:** 1 Department of Imaging, IRCCS Bambino Gesù Children’s Hospital, Piazza Di Sant’Onofrio 4, 00165 Rome, Italy; 2 Department of Clinical Medicine, the Artic University of Norway, Tromsø, Norway; 3 Department of Clinical Radiology, Great Ormond Street Hospital for Children NHS Foundation Trust, Great Ormond Street, London, UK; 4 Great Ormond Street Hospital for Children, UCL Great Ormond Street Institute of Child Health, London, UK; 5 NIHR Great Ormond Street Hospital Biomedical Research Centre, Bloomsbury, London, UK; 6 Department of Radiology, St. George’s Hospital, London, UK; 7 Rheumatology Division, IRCCS, Bambino Gesù Children’s Hospital, Rome, Italy; 8 Clinica Pediatrica E Reumatologia, IRCCS Istituto Giannina Gaslini, Genoa, Italy; 9 Department of Neurosciences, Rehabilitation, Ophthalmology, Genetic and Maternal Infantile Sciences (DINOGMI), University of Genoa, Genoa, Italy; 10 Divisione Radiologia, IRCCS Istituto Giannina Gaslini, Genoa, Italy; 11 Department of Radiology, University Hospital of North Norway, Tromsø, Norway

**Keywords:** Adolescents, Children, Hip, Inflammation, Joint damage, Juvenile idiopathic arthritis, Magnetic resonance imaging, Scoring system, Young adults

## Abstract

**Background:**

Hip involvement predicts severe disease in juvenile idiopathic arthritis (JIA) and is accurately assessed by MRI. However, a child-specific hip MRI scoring system has not been validated.

**Objective:**

To test the intra- and interobserver agreement of several MRI markers for active and chronic hip changes in children and young adults with JIA and to examine the precision of measurements commonly used for the assessment of growth abnormalities.

**Materials and methods:**

Hip MRIs from 60 consecutive children, adolescents and young adults with JIA were scored independently by two sets of radiologists. One set scored the same MRIs twice. Features of active and chronic changes, growth abnormalities and secondary post-inflammatory changes were scored. We used kappa statistics to analyze inter- and intraobserver agreement for categorical variables and a Bland–Altman approach to test the precision of continuous variables.

**Results:**

Among active changes, there was good intra- and interobserver agreement for grading overall inflammation (kappa 0.6–0.7). Synovial enhancement showed a good intraobserver agreement (kappa 0.7–0.8), while the interobserver agreement was moderate (kappa 0.4–0.5).

Regarding acetabular erosions on a 0–3 scale, the intraobserver agreement was 0.6 for the right hip and 0.7 for the left hip, while the interobserver agreement was 0.6 for both hips. Measurements of joint space width, caput–collum–diaphyseal angle, femoral neck–head length, femoral width and trochanteric distance were imprecise.

**Conclusion:**

We identified a set of MRI markers for active and chronic changes in JIA and suggest that the more robust markers be included in future studies addressing clinical validity and long-term patient outcomes.

**Supplementary Information:**

The online version contains supplementary material available at 10.1007/s00247-022-05502-8.

## Introduction

Juvenile idiopathic arthritis (JIA) is the most common chronic rheumatologic disease in children [1]. It comprises a group of clinically heterogeneous arthritides of unknown origin that develop before the age of 16 years and persist for at least 6 weeks. The disease is characterized by a chronic inflammatory process of the synovium and periarticular tissue that can lead to structural damage and growth abnormalities. The incidence of JIA varies from 1.6 to 23 in 100,000, with a prevalence of 3.8–400 in 100,000 [2].

Hip involvement is common in children with JIA, occurring in approximately 20–50% of cases [3] and is considered a predictor of severe disease, carrying a high risk of disability. Typically, both hips are affected, but unilateral involvement is occasionally seen. The majority of children with active hip disease develop irreversible changes within 5 years of diagnosis [4] and approximately 26–44% require a total hip replacement within the first 10 years of disease onset [5].

Early detection and targeted treatment of active disease is essential to improve long-term outcomes [6]. Although most children with JIA have ongoing disease into adulthood, it has been suggested that there is a “window of opportunity” during the early, subclinical stage, where prompt instigation of treatment might delay progression and induce remission [7, 8]. Unfortunately, clinical assessment of the hips, even when performed by experienced rheumatologists, is difficult because the hip joint is deep-lying and not easy to palpate. There is thus a need for accurate diagnostic tools to depict both active and chronic JIA-related joint changes, as has been highlighted in several studies [6, 9].

For adults with rheumatoid arthritis, several validated scoring systems for hip involvement exist, including the Hip Inflammation Magnetic Resonance Imaging Scoring System (HIMRISS) and Hip Osteoarthritis MRI Scoring System (HOAMS) [10, 11]. For children, however, although several scoring systems have been proposed [12–16], no child- and hip-specific scoring system including both inflammatory and permanent changes has been validated [17]. In one study including 79 children with hip involvement, of whom 22 had confirmed JIA, the inter- and intraobserver agreement of several selected parameters were addressed using a simplified MR-based scoring system on a 0–1 scale [14]. The most reliable features were the presence of joint effusion, bone marrow edema and the subjective assessment of synovium [14]. The study reported significant differences across parameters in the intraobserver reliability and a poor–moderate interobserver reliability for most parameters [14]. However, the study was limited to the active inflammatory domain. Moreover, as underlined by the authors, most children with JIA did not have a confirmed inflammatory disease, further weakening the robustness of the study [14]. Other authors have compared MRI and clinical or laboratory findings in small datasets, thus without testing the precision of the MRI markers applied [12, 13, 15, 16].

More recently, two papers addressing non-enhanced MRI in the diagnosis of hip changes in JIA were published — one retrospective observational study including 97 children with clinically suspected hip JIA [18] and one study on whole-body MRI for the quantification of a total inflammatory joint score [19]. However, again, repeatability studies of the suggested scores were not performed.

Our study is a first step toward establishing a robust MRI-based scoring system for active and chronic JIA changes of the hip to be used for monitoring treatment effect in daily practice as well as measuring outcomes in clinical trials. We tested the intra- and interobserver agreement of a set of MRI markers for active and chronic hip changes and examined the precision of measurements commonly used for assessing growth abnormalities.

## Materials and methods

This study is part of a large longitudinal multicenter project (Health-e-Child) aimed to establish imaging-based scoring systems for children and adolescents with JIA with wrist or hip involvement. Leading pediatric musculoskeletal radiologists and clinical rheumatologists at four centers — Bambino Gesù Children’s Hospital (OPBG), Rome; Giannina Gaslini Institute (IGG), Genoa; Hopital Necker Enfant Malades (HNEM), Paris; and Great Ormond Street Hospital (GOSH), London — were involved in devising an MRI-based scoring system for JIA. The present project was approved by the institutional research ethics committee at OPBG and IGG. Written informed consent was obtained from all the patients or their caregivers. For the purpose of this particular study, we included 60 consecutive children, adolescents and young adults over a 2-year interval with a diagnosis of JIA and confirmed or suspected hip involvement (37 studied at OPBG and 23 at IGG) according to the International League of Associations for Rheumatology (ILAR) classification [20]. Children and adolescents of any disease severity and activity level were included, irrespective of current or previous medical treatments. All patients underwent MRI without sedation.

### Magnetic resonance imaging protocol

All MRI examinations were performed on a 1.5-tesla (T) MRI system (Achieva Intera; Philips Medical Systems, Best, The Netherlands), using a body coil. The field-of-view included the whole pelvis to allow visualization of both hips. The children were imaged supine with the legs straight and the feet in a neutral position.

The following sequences were acquired:

- Three-dimensional (3-D) T1-weighted turbo spin-echo (TSE) sequence with repetition time/echo time (TR/TE) 600/10 ms, acquired and reconstructed voxel size of 1 × 1 × 1 mm, number of signal averages 2, acquisition time about 5 min;
- T2-weighted TSE fat-saturated (FS) sequence with TR/TE 4,400/70 ms, voxel size 0.55 × 0.69 × 3 mm, base resolution 218, section thickness/gap 3/0.3 mm, number of signal averages 1, acquisition time about 4 min;
- 3-D spoiled gradient echo (GRE) FS sequence with TR/TE 40/7 ms, flip angle (FA) 25°, voxel size 1 × 1 × 1 mm, acquisition time about 4 min, acquired immediately (“early”) and approximately 5 min (“late”) after manual injection of 0.2 mL/kg of gadoteric acid 0.5 mmol/m (Dotarem; Guerbet, Roissy, France) through a 21-gauge (G) cannula inserted into an arm vein, followed by a flush of 10 mL saline.

All sequences were acquired in the coronal plane. The mean imaging time, including the time for positioning and injection, was approximately 25 min.

### Scoring

Prior to scoring, we conducted three calibration sessions lasting 2 days each, using 30 MRI cases not included for analysis in this study to ensure standard terminologies and definitions could be agreed upon. We used an imaging atlas with relevant examples of each variable and grade as a reference to help maintain a consistent standard of scoring across all readers (see Online Supplementary Material 1 for the scoring system and Online Supplementary Material 2 for imaging atlas).

All hip MRIs were scored by two sets of radiologists. The first set scored the same MR images twice (the second time after a wash-out period of 3 weeks). In this set, the scoring was performed in consensus by one pediatric radiologist (L.T.dH., with 14 years of experience) and one of two additional pediatric radiologists (P.L.D.P., with 9 years of experience, or P.dA., with 5 years of experience) at OPBG. The second set included one pediatric radiologist (S.C.S., with 7 years of experience) at GOSH, who scored all the MRI images once independently. All radiologists were blinded to disease duration, clinical symptoms and findings, JIA subtype and prior imaging.

### Inflammatory changes

**Fig. 1:**
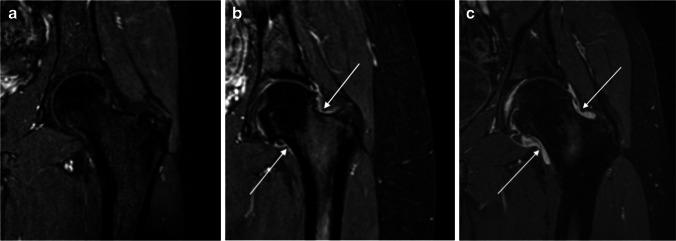
Degree of post-contrast synovial enhancement of the left-hip MRIs in three children with juvenile idiopathic arthritis (JIA), demonstrated on coronal three-dimensional (3-D) gradient echo MRI sequences with fat saturation. **a** No visible synovial enhancement (score 0) in a 17-year-old boy. **b** Mildly increased enhancement (*arrows*) (score 1) in a 16-year-old girl. **c** Severely increased enhancement (*arrows*) (score 2) in a 15-year-old boy

**Fig. 2:**
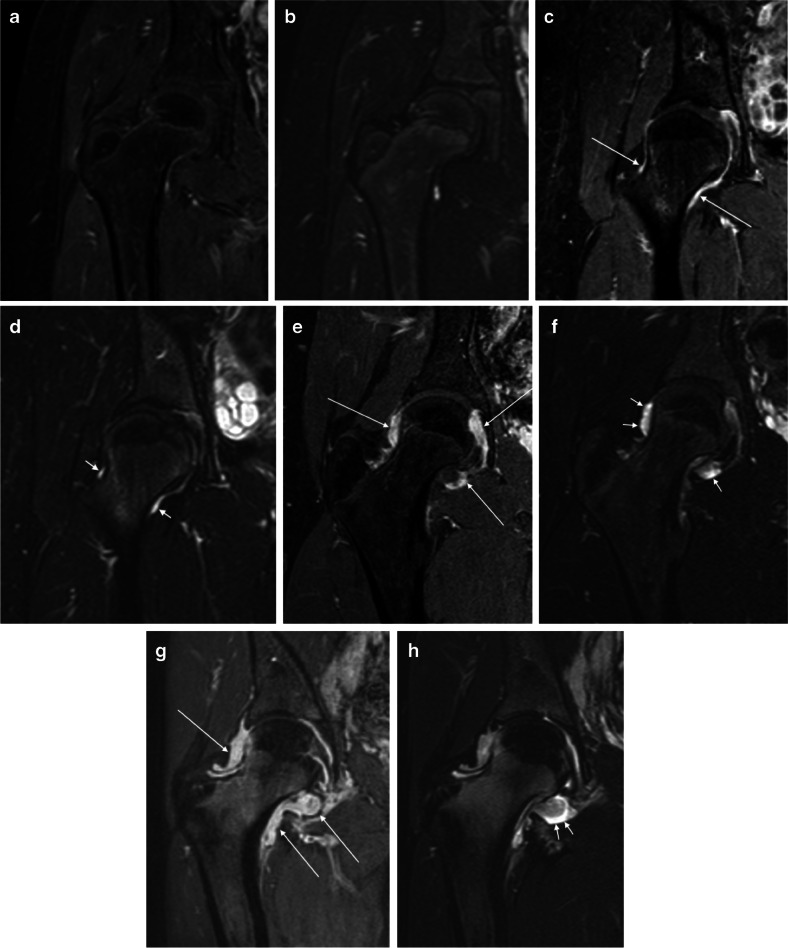
Overall degree of inflammation in right-hip MRIs in four pairs of images from four children with juvenile idiopathic arthritis (JIA) across different levels of severity. All images are demonstrated using coronal post-contrast three-dimensional (3-D) gradient echo with fat saturation (**a, c, e, g**) and coronal fat-saturated T2-weighted turbo spin-echo (**b, d, f, h**) MRI sequences. **a, b** No inflammation (score 0) in a 12-year-old boy. **c, d** Mild synovial thickening with moderate increase in post-contrast enhancement (*long arrows*) and sliver of effusion (*short arrows*) (score 1) in a 16-year-old girl. **e, f** Moderate synovial thickening with moderately increased post-contrast enhancement (*long arrows*) and mild effusion (*short arrows*) (score 2) in a 15-year-old boy. **g, h** Severe synovial thickening and increased post-contrast enhancement, more evident at the medial aspect of the joint (*long arrows*), with mild/moderate effusion (*short arrows*) (score 3) in a 17-year-old girl

**Fig. 3:**
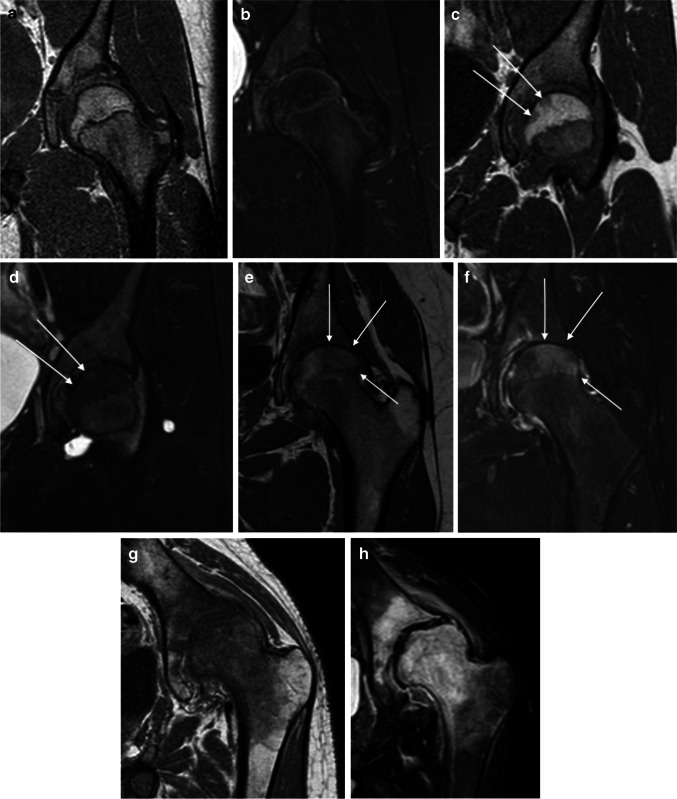
Femoral head bone marrow edema demonstrated on MRI of the left hip in four pairs of images from four children with juvenile idiopathic arthritis (JIA) across different severity levels. Bone marrow edema was defined as hypointense areas on T1-W sequences with corresponding hyperintense areas on fat-saturated T2-W sequences in the bone marrow. All images are demonstrated using coronal three-dimensional (3-D) T1-weighted turbo spin-echo (**a, c, e, g**) and fat-saturated T2-weighted turbo spin echo (**b, d, f, h**) MRI sequences. **a, b** No visible bone marrow edema (score 0) in a 13-year-old boy. **c, d** Two focal areas of bone marrow edema (less than 33% of the bone volume, *arrows*) (score 1) in a 11-year-old girl. **e, g** Large area of bone marrow edema (between 33 and 66% of the bone volume, *arrows*) (score 2) in a 14-year-old girl. **g, h** Widespread bone marrow edema (almost 100% of the bone volume) (score 3) in a 15-year-old boy

Based on the pre- and late post-contrast 3-D GRE and the coronal T2-W FS images, we scored:

1. Synovial enhancement intensity (using different scoring scales, Fig. 1) and synovial thickening (measured both subjectively and objectively);
2. Presence of effusion;
3. Degree of overall synovial inflammation including thickening and enhancement intensity;
4. Degree of overall inflammation (Fig. 2), adding effusion to the degree of overall synovial inflammation;
5. Bone marrow edema, which was defined as an area of high signal intensity on T2-W FS images with corresponding low signal intensity on T1-W images and was assessed in the femoral head based on the proportion of bone involved (volume) (Fig. 3), in the acetabulum (measured subjectively) and in the femoral neck as absent or present (0/1).

### Structural joint damage

**Fig. 4:**
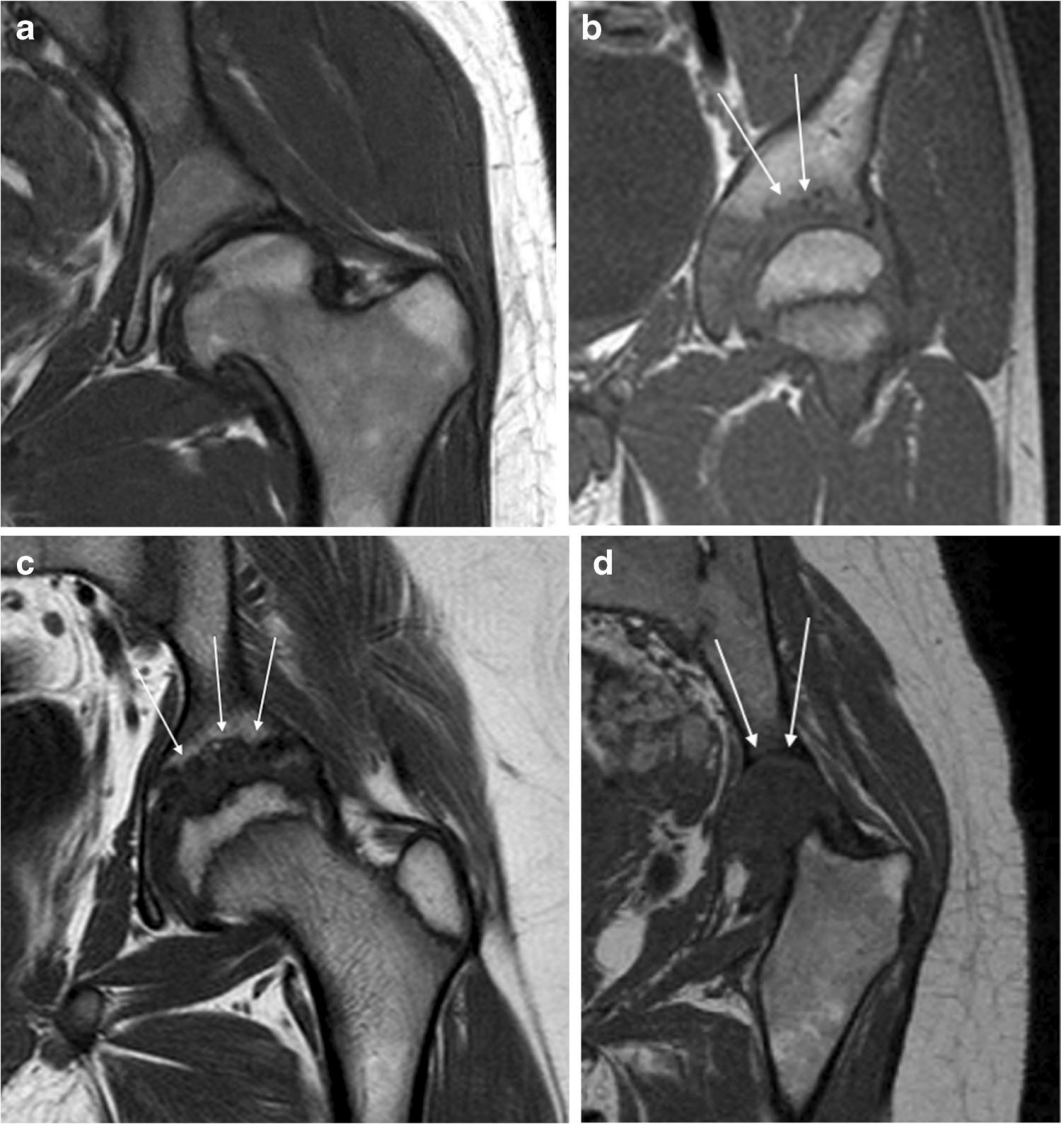
Erosions demonstrated on MRI at the left acetabulum as shown on coronal three-dimensional (3-D) T1-weighted turbo spin-echo sequences in patients with juvenile idiopathic arthritis (JIA). **a** No visible acetabular erosions (score 0) in a 20-year-old woman. **b** Some erosions on the superior aspect of the acetabulum (< 33% of the surface, *arrows*) (score 1) in a 18-year-old man. **c** Multiple acetabular erosions (between 34% and 66% of the surface, *arrows*) (score 2) in a 13-year-old boy. **d** Erosive changes of the whole acetabular surface (*arrows*) (score 3) in a 19-year-old woman with complete destruction of the femoral head (*arrows*)

**Fig. 5:**
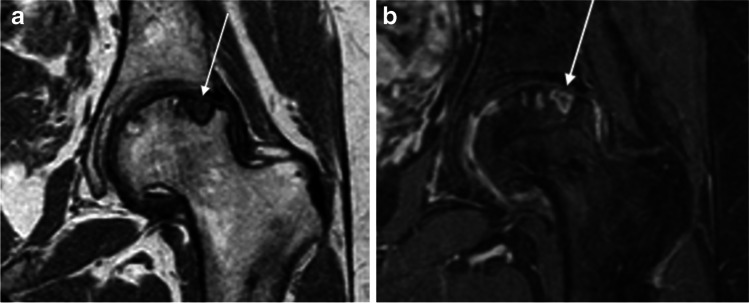
Left-hip MRI of active erosion in an 18-year-old woman with juvenile idiopathic arthritis (JIA). **a, b** Coronal three-dimensional (3-D) T1-weighted turbo spin-echo (**a**) and post-contrast 3-D gradient echo with fat saturation (**b**). These images show an active erosion (erosion filled with enhancing pannus) (*arrows*)

Structural joint damage was evaluated on the 3-D TSE T1-W images, using fluid-sensitive and post-contrast images when appropriate. The following features were evaluated and scored:

1. Erosion (defined as a bony depression seen on at least two planes) in the femoral head based on the proportion of head volume involved, in the femoral neck as absent or present (0/1) and in the acetabulum (Fig. 4). Active erosions (defined as an erosion filled with enhancing pannus) were scored in the femoral head (Fig. 5).
2. Flattening of the femoral head was assessed in the coronal plane (mid-section) compared to what is expected for age, first subjectively and thereafter using a Mose circle. Bone cysts were described as sharply delineated, enhancing lesions with high signal on fluid-sensitive sequences and were scored as absent/present in three locations (femoral head, neck and acetabulum).

### Cartilage damage

Based on 3-D GRE T1-W sequences, we assessed the joint cartilage width superiorly (mid-weight-bearing area), first judging it subjectively to be normal, mildly, moderately or severely narrowed and then taking measurements in millimeters. We also evaluated and scored cartilage in terms of signal abnormalities and morphological changes, as well as symmetry (right versus left joint space width). Based on the coronal T1-W sequences, we measured femoral neck width (in mm), femoral head/neck length (in mm), caput–collum–diaphyseal (CCD) angle and trochanteric femoral head distance (in mm). We also evaluated whether the physis was patent; the presence of coxa magna, coxa brevis or protrusio acetabuli; and the presence of fovea enlargement. Finally, we evaluated the presence of osteophytes and sclerosis on both coronal T1-W and fluid-sensitive FS sequences.

### Statistical analysis

Continuous data are presented as means (± standard deviation [SD]), ordinal data as medians (ranges) and dichotomous data as proportions. We analyzed intra- and interobserver agreement using a simple or a weighted (linear) Cohen kappa coefficient with 95% confidence interval. A kappa score of < 0.2 was considered poor, 0.21–0.40 fair, 0.41–0.60 moderate, 0.61–0.80 good and 0.81–1.00 very good [21]. We analyzed differences in measurements using 95% limits of agreement (termed repeatability coefficient, when used for repeat measurements) as per Bland–Altman. Bland–Altman plots are generally interpreted informally and a clinically acceptable agreement was set at 15% [22]. A significance level of 0.05 was decided a priori and all the reported *P-*values are two-tailed. Statistical analyses were performed using SPSS Statistics, version 27 (IBM, Armonk, NY).

## Results

**Fig. 6:**
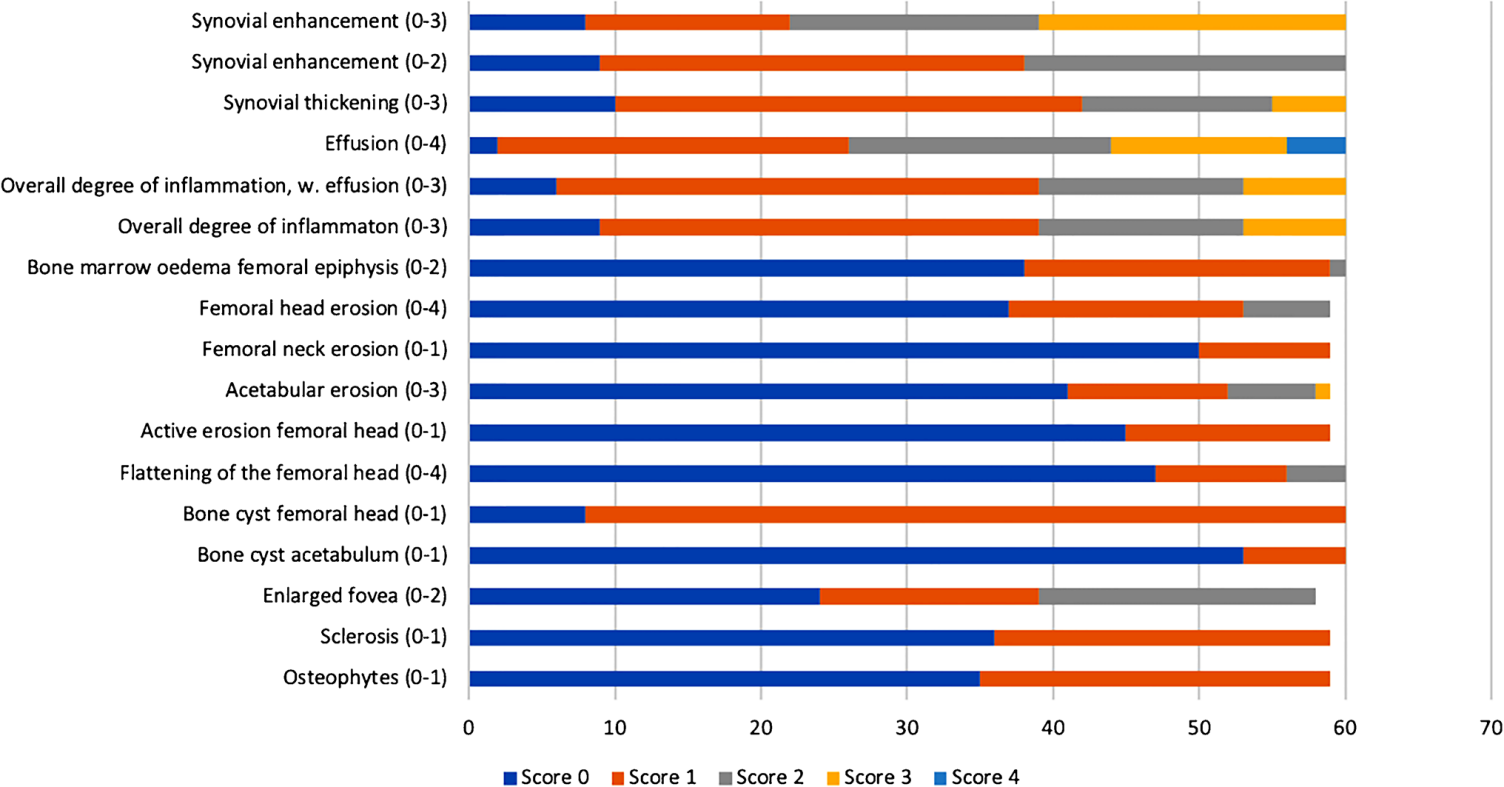
Distribution of MRI scores for the various features assessed in 60 children through young adults with juvenile idiopathic arthritis (JIA) in this study (right hip, observer 1, second reading). The x-axis shows the number of examined hips

We included 60 MRIs from 60 children and young adults with JIA (35 female) and confirmed or suspected hip involvement, with a mean age 14.9 years (range 5.5–20 years). Of these, 23 had oligoarticular JIA (18 oligoarticular-extended and 5 oligoarticular-persistent), 17 had the polyarticular JIA subtype, 8 had enthesitis-related arthritis, 10 systemic JIA, 1 psoriatic JIA and 1 undifferentiated JIA. The mean disease duration at the time of the MRI was 8.6 years (range 0.2–18 years). The distribution of changes seen for the right hip is shown in Fig. 6.

Table 1 shows the agreement within and between readers for the assessment and grading of inflammatory and structural JIA changes as examined on MRI for right and left hips, separately.

**Table 1:** Test–retest analysis of features used to describe inflammatory and chronic changes on MRI in 60 children through young adults (35 females) ages 6–20 years with juvenile idiopathic arthritis and hip involvement

Type of damage	Right hip		Left hip	
	Intra-reader kappa (95% CI)	Inter-reader kappa (95% CI)	Intra-reader kappa (95% CI)	Inter-reader kappa (95% CI)
Inflammatory domain				
Synovial enhancement (0–3)	0.7 (0.6–0.9)	0.3 (0.2–0.4)	0.7 (0.5–0.8)	0.4 (0.2–0.5)
Synovial enhancement (0–2)	0.8 (0.6–0.9)	0.4 (0.2–0.6)	0.7 (0.5–0.8)	0.5 (0.3–0.7)
Synovial enhancement (0–1)	0.7 (0.4–1.0)	0.2 (–0.1–0.5)	0.5 (0.0–0.9)	0.1 (–0.2–0.5)
Synovial thickening subjective (0–3)	0.9 (0.8–1.0)	0.5 (0.3– 0.7)	0.8 (0.6– 0.9)	0.4 (0.2–0.6)
Effusion (0–4)	0.6 (0.4–0.8)	0.4 (0.2–0.6)	0.7 (0.5–0.8)	0.3 (0.2–0.4)
Overall synovial inflammation (0–3)	0.8 (0.6–1.0)	0.4 (0.2–0.6)	0.7 (0.5–0.9)	0.4 (0.3–0.6)
Overall degree of inflammation, including effusion (0–3)	0.7 (0.5–0.9)	0.6 (0.4–0.7)	0.7 (0.5–0.9)	0.6 (0.4–0.7)
Bone marrow edema				
Femoral epiphysis (0–2)	0.7 (0.5–0.9)	0.4 (0.1–0.6)	0.7 (0.5–0.9)	0.3 (0.1–0.6)
Acetabulum (0–1)	0.7 (0.6–0.9)	0.2 (0.0–0.4)	0.7 (0.5–0.9)	0.3 (0.1–0.4)
Femoral neck (0–1)	0.7 (0.5–0.9)	0.3 (0.0–0.5)	0.7 (0.6–0.9)	0.3 (0.0–0.5)
Structural bone damage domain				
Erosion				
Femoral head (0–3)	0.7 (0.5–0.9)	0.4 (0.2–0.6)	0.8 (0.6–0.9)	0.5 (0.3–0.7)
Femoral neck (0–1)	0.8 (0.5–1.0)	0.2 (0.1–0.6)	0.7 (0.5–0.9)	0.1 (0.1–0.4)
Acetabulum (0–3)	0.6 (0.4–0.8)	0.6 (0.4–0.8)	0.7 (0.5–0.8)	0.6 (0.4–0.8)
Acetabulum (0–2)	0.6 (0.4–1.0)	0.3 (0.1–0.5)	0.7 (0.5–0.8)	0.4 (0.2–0.6)
Active erosion femoral head (0–1)	0.9 (0.8–1.0)	0.6 (0.3–0.9)	0.9 (0.7–1.0)	0.6 (0.4–0.9)
Flattening of the femoral head^a^ (0–4)	0.7 (0.5–0.9)	0.4 (0.2–0.6)	0.6 (0.4–0.8)	0.3 (0.1–0.5)
Flattening femoral head Mose^b^ (0–4)	0.7 (0.5–0.9)	0.3 (0.1–0.5)	0.6 (0.4–0.8)	0.3 (0.1–0.5)
Bone cyst (0–1)				
Femoral head	0.9 (0.6–1.0)	0.7 (0.4–1.0)	0.8 (0.6–1.0)	0.7 (0.5–1.0)
Acetabulum	0.8 (0.5–1.0)	0.3 (0.0–0.7)	0.8 (0.5–1.0)	0.6 (0.3–0.9)
Enlarged fovea (0–2)	0.7 (0.6–0.9)	–0.1 (0.2–0.1)	0.8 (0.6–0.9)	0.1 (0.1–0.2)
Sclerosis (0–1)	0.8 (0.6–0.9)	0.1 (0.1–0.2)	0.8 (0.7–1.0)	0.1 (0.0–0.2)
Osteophytes (0–1)	0.9 (0.8–1.0)	0.0 (0.0–0.2)	0.9 (0.8–1.0)	0.2 (0.0–0.4)
Joint space width (0–3)	0.7 (0.6–0.9)	0.2 (0.0–0.4)	0.6 (0.5–0.8)	0.2 (0.1–0.4)
Cartilage changes (0–4)	0.4 (0.3–0.6)	0.3 (0.1–0.4)	0.4 (0.2–0.6)	0.3 (0.1–0.4)

*CI* confidence interval

^a^Flattening of femoral head as assessed subjectively

^b^Flattening of femoral head as assessed using a Mose circle

### Inflammatory domain

There was a good intra- and interobserver agreement (with a kappa value of 0.7 and 0.6, respectively) for grading overall impression of inflammation on a 0–3 scale (effusion included). Similarly, grading overall impression of inflammation, omitting effusion, performed well, with an intraobserver kappa of 0.7–0.8 and an interobserver kappa of 0.4 (Table 1).

Grading synovial enhancement performed best on a 0–2 scale, with a good intraobserver and a moderate interobserver agreement (kappa of 0.7–0.8 and 0.4–0.5, respectively). The intraobserver agreement for subjective evaluation of synovial thickening was good to very good (kappa of 0.8–0.9) while the interobserver agreement was moderate (0.4–0.5) (Table 1).

There was good intraobserver agreement for grading effusion with kappas of 0.6–0.7, while the interobserver agreement for the same variable was fair (kappa 0.3–0.4). Regarding bone marrow edema, the intraobserver agreement was good, with a kappa value 0.7 for all locations bilaterally (femoral epiphysis, acetabulum and femoral neck); however, the interobserver kappa values were poor, ranging between 0.2 and 0.4 (Table 1).

### Structural damage domain

There was a good intra- and interobserver agreement for grading erosions in the acetabulum on a 0–3 scale, with kappa values of 0.6–0.7 and 0.6, respectively. Regarding the grading of femoral head erosions, the intraobserver agreement was highly satisfactory (kappa values of 0.7–0.8), while the interobserver agreememt was moderate (kappa 0.4–0.5) (Table 1).

There was an excellent intraobserver agreement for grading active erosions of the femoral head, with kappa values of 0.9 and the interobserver agreement was good with a kappa of 0.6. The kappa values for the femoral head flattening with or without the use of a Mose circle performed well for the same observer (kappa value of 0.7 for the right hip and 0.6 for the left hip), while agreement was significantly lower between observers (0.3–0.4).

There was only one cyst in the femoral neck, thus we could not estimate a kappa value.

The agreement for bone cyst on the femoral head and acetabulum, enlarged fovea, sclerosis and osteophytes is listed in Table 1.

Measurement of joint space width evaluated in millimeters performed poorly, with wide 95% limits of agreement (LOA) ranging from –1.6 to 2.0 mm for the intraobserver and from –2.7 to 3.3 mm for the interobserver values, corresponding to 129% and 214% of the mean value, respectively (Table 2). Kappa values for the subjective assessment of joint cartilage width on a 0–3 scale were good, ranging from 0.6 to 0.7 for the same observer, while the interobserver agreement was poor, with a kappa value of 0.2. The agreement for signal abnormalities/morphological changes on a 0–4 scale was fair (kappa values of 0.3–0.4) (Table 1).

**Table 2:** Test–retest analysis of features used in evaluation of growth changes on MRI in 60 children with juvenile idiopathic arthritis and hip involvement (right hip)

Markers for assessment of growth	OBS 1		Intraobserver (OBS1) (1^st^ vs. 2^nd^ measurement)		OBS 2	Interobserver (OBS 1 vs. OBS 2)	
	1^st^ mean (SD)	2^nd^ mean (SD)	Mean diff. (SD)	95% LOA	Mean (SD)	Mean diff. (SD)	95% LOA
Femoral neck width, mm	29.0 (14.9)	25.0 (4.2)	4.4 (14.6)	–24.8 to 33.6	28.2 (8.4)	3.8 (7.5)	–11.2 to 18.8
Femoral head/neck length, mm	72.3 (19.6)	76.3 (13.6)	3.1 (14.4)	–25.7 to 31.9	26.4 (5.4)	3.8 (7.5)	–11.2 to 18.8
CCD angle, degrees	132.0 (7.5)	131.1 (8.6)	0.5 (6.8)	–13.1 to 14.1	132.9 (5.5)	1.5 (8.3)	–15.1 to 18.1
TFHD, mm	18.4 (5.8)	18.3 (5.9)	0.1 (2.8)	–5.5 to 5.7	22.6 (11.0)	0.1 (2.8)	–3.5 to 5.7
Joint space width, mm	2.8 (1.3)	3.0 (1.5)	0.2 (0.9)	–1.6 to 2.0	3.3 (1.3)	0.3 (1.5)	– 2.7 to 3.3

*CCD* caput–collum–diaphyseal angle*, diff.* difference, *LOA* limits of agreement, *OBS* observer, *SD* standard deviation, *TFHD* trochanteric–femoral head distance

### Markers for the assessment of growth

Measurements of the CCD, femoral head–neck length, femoral neck width and trochanteric femoral head distance were imprecise, with a wide 95% LOA (Table 2).

## Discussion

This study is part of a larger project to establish MRI markers for active and chronic disease in children, adolescents and young adults with JIA with hip involvement. In this substudy we tested numerous markers (isolated and in combination) to identify those that are sufficiently robust to be included in a future MRI scoring system. The study is novel in that it provides the precision of various MR imaging biomarkers for both inflammatory and chronic changes in children and adolescents with JIA-related hip involvement. One previous paper addressed the accuracy of a simplified MR score for assessing active changes, reporting a variable intraobserver agreement across both observers and parameters, ranging from poor to excellent, while the interobserver agreement was consistently moderate for effusion and marrow edema and less satisfactory for other parameters [14].

We have identified a set of MRI markers for hip involvement in children and adolescents with JIA. The more precise inflammatory markers include overall degree of inflammation on a 0–3 scale, synovial enhancement on a 0–2 scale and active erosions on a 0–1 scale, while assessment of bone marrow edema performed well for the same-observer only. For structural bone damage, grading of femoral head and acetabular erosions performed well. Direct measurements were imprecise.

Surprisingly, our study showed that grading of synovial enhancement on a 0–1 scale performed poorer than grading based on 0–2 and 0–3 scales. This is most likely a result of the difficulties in setting a precise cut-off between physiological synovial enhancement and mildly increased enhancement suggestive of synovial inflammation. In contrast to the study by Porter-Young et al. [14], in our population very few cases were scored as non-enhancing, thus yielding a skewed dataset for the kappa analysis. Indeed, the lack of a precise cut-off is a diagnostic challenge in that it can lead to both overdiagnosis with unnecessary treatment, and underdiagnosis with an increased risk of structural damage and poorer long-term outcome. This underscores the need for prospective studies establishing reference standards across ages.

Another challenge in grading synovial enhancement is timing of the post-contrast images. Previous studies have shown that timing strongly influences the degree of synovial enhancement in the assessment of both wrists [23] and knees [24]. Despite the increasing use of MRI in arthritis, there is no consensus on the exact timing for post-contrast images, the suggested interval being within 5 min [25]. The rationale behind early post-contrast images is that, if acquisition is delayed too long, contrast washout from the synovium into the joint fluid obscures the borders between synovium and an effusion, as was demonstrated in two studies of patients with rheumatoid arthritis [26, 27]. Thus, a standardized protocol is crucial for follow-up of known pathology, and also for clinical trials across institutions. In the present study, we acquired post-contrast sequences approximately 5 min after the contrast injection.

Regarding bone marrow edema, despite good intraobserver agreement, the interobserver agreement was disappointing and not in line with a previous study on wrist MRI [28]. We speculate that the size and shape of the scored volumes might play a role because carpal bones are significantly smaller than hips, thus fewer slices are included for assessment.

We have possible explanations for the unsatisfactory interobserver agreement for some of the other features. Among the structural damage markers, we believe that the agreement for cartilage lesions was poor for two main reasons. First, the acetabular and femoral layer of articular cartilage is very thin in the hip joint and it is extremely difficult to reliably distinguish between partial- and full-thickness lesions. Second, in the growing child the cartilage becomes thinner with time, thus it is challenging to differentiate the physiological thickness reduction caused by growth from the presence of pathological erosions. Future studies comparing our data with the MRI data obtained from healthy children could help elucidate this matter. Moreover, osteophytes were present only in few patients, and this might have affected the suboptimal results.

Last, further calibrations could improve the inter-reader reliability. The poor results for direct measurements as the CCD, femoral neck–head length, femoral width and trochanteric distance were expected and in line with previous studies [29].

Of note is the excellent agreement for assessing active erosions, both within and between observers. Moreover, the assessment of acetabular erosions was precise, as was the assessment of femoral head and neck erosions for the same reader. Whether MRI might replace conventional radiographs, however, remains to be addressed.

Our study has some limitations. First, there is the subjective nature of any MRI scoring system, with differences in measurements and inherent biases caused by different radiologists’ experience and understanding of the factors required to score, although we tried to alleviate this with calibration sessions and the use of an imaging atlas. Moreover, some of the features evaluated in our scoring system were extremely rare (i.e. cyst on femoral neck, coxa brevis), thus it was not possible to assess the agreement for those variables.

The strengths of the study are large sample size covering a wide spectrum of pathological changes, within both the inflammatory and the bone damage domains. Furthermore, we used state-of-the-art MRI protocols across two centers, both including intravenous contrast agent, and our scan parameters were selected to provide the best images within a reasonably short scan time. Last, we performed meticulous calibration sessions prior to the scoring sessions, preceded by a pilot study and an atlas, to ensure that readers could interpret imaging findings in a consistent manner.

In a next paper we plan to complete the validation process of the present MRI scoring system by testing its clinical validity and responsiveness to change, aiming to present a final MRI scoring system to be used as a primary outcome measure in clinical trials with the purpose of evaluating the efficacy of novel antirheumatic drugs for JIA similar to that already established for rheumatoid arthritis (RA) in adults [30–33]. Once obtained, this scoring system might be usefully employed in several settings in JIA patients. Particularly, it could be used prior to therapy to identify children who need more aggressive treatment and during the pharmacological treatment to monitor its efficacy and to assess more accurately their remission status. Moreover, it has been recently reported that persistent synovitis on hip MRI in children with JIA in clinical remission predicts disease flare [34]. Therefore, our scoring system could be extremely helpful in children in clinical remission where the depiction of a silent synovitis on MRI might allow prompt treatment with a possible considerable improvement of disease progression.

## Conclusion

This work is a first step toward establishing a valid MRI scoring system for JIA-related hip changes. Several of the MRI markers for both active and chronic changes showed a high reproducibility, the most interesting being the overall synovial inflammation and the evaluation of active erosions. We suggest that the more robust variables be used in future studies assessing clinical validity, responsiveness to change and long-term patient outcomes.

## Supplementary Information

Below is the link to the electronic supplementary material.

Supplementary file1 (DOCX 36.6 KB) Online Supplementary Material 1 Hip MRI scoring system for juvenile idiopathic arthritis (JIA)

Supplementary file2 (PDF 13535 KB) Online Supplementary Material 2 Imaging atlas with relevant examples of each variable and grade in juvenile idiopathic arthritis (JIA)
