# Supplementary material for: A novel magnetic resonance imaging scoring system for active and chronic changes in children and adolescents with juvenile idiopathic arthritis of the hip
Source: Pediatr Radiol. 2022 Sep 23;53(3):426–37. doi: 10.1007/s00247-022-05502-8 (PMC9968695; doi:10.1007/s00247-022-05502-8)
Supplement: Supplementary file 1 — Supplementary file1 (DOCX 36.6 KB) Online Supplementary Material 1 Hip MRI scoring system for juvenile idiopathic arthritis (JIA) [file 247_2022_5502_MOESM1_ESM.docx]

| **SCORING OF HIP MRI FOR JIA** |
| --- |
| **SYNOVITIS** (see atlas)  Based on (late*) post-contrast 3-D GRE images (if present) and coronal T2-W FS images. Synovitis is first scored according to enhancement and second to the degree of synovial thickening and third to the degree of overall inflammatory changes (enhancement/synovial thickness) and finally to the degree of overall inflammation (including effusion). |

**Online Supplementary Material 1:** Hip MRI Scoring System for JIA

| **1. Enhancement intensity** (as compared to pre-contrast FS images. In cases of patchy increased enhancement, the predominant appearance should be scored). First, at a 0–3 scale:  Score 0: no, or very subtle synovial enhancement  Score 1: mildly increased synovial enhancement  Score 2: moderately increased synovial enhancement (SI<vessels)  Score 3: severely increased synovial enhancement (SI≥vessels)  And second, at a 0–2 scale (=wrist score):  Score 0: no, or very subtle synovial enhancement  Score 1: mildly increased synovial enhancement (more than muscle, less than vessel)  Score 2: moderate to severely increased synovial enhancement (≥vessel)  **2. Assessment of the synovium** (based on post-contrast image)   1. **Thickening of the synovium** (one layer) as:   0 = no thickening  1 = mild thickening  2 = moderate  3 = severe   1. **Thickening of the synovium** (in mm) measured medially and laterally on coronal images (mid-section). In case one cannot differentiate between the layers, measure all the capsule–osseous distance and divide in two. Add measurement on axial reconstructed images (only for OPBG cases). 2. **Enhancement no/yes**: 0/1   **3. Overall synovial inflammation** (both enhancement intensity and synovial thickness) (at a 0–3 scale):  Score 0: no  Score 1: mild overall involvement  Score 2: moderate overall involvement Score 3: severe overall involvement  **4. Effusion** (based on T2-W FS images)  0 = no  1 = a sliver  2 = mild  3 = moderate amount  4 = large amount of fluid  **5. Overall degree of inflammation** (=wrist score, i.e. enhancement intensity, synovial thickness and effusion):  Score 0: no inflammation  Score 1: mild inflammation  Score 2: moderate inflammation  Score 3: severe inflammation | | | | | | | | | | | | | | | |
| --- | --- | --- | --- | --- | --- | --- | --- | --- | --- | --- | --- | --- | --- | --- | --- |
| **SYNOVITIS SCORING** | | | | | | | | | | | | | | | |
|  | Enhancement intensity | | | Synovial thickness | | | | | Synovial  enhancement | | Overall synovial inflammation | | Effusion | Overall degree of inflammation | |
|  | 0–3 | 0–2 | | 0–3 | | In mm  M L | | | 0–1 | | 0–3 | | 0–4 | 0–3 | |
| Rt hip |  |  | |  | |  | |  |  | |  | |  |  | |
| Lt hip |  |  | |  | |  | |  |  | |  | |  |  | |
|  | | | | | | | | | | | | | | | |
| **BONE MARROW CHANGES (BMC)**  Defined as high signal intensity on T2-W FS/STIR images with corresponding low SI on T1-W images. Assess BMC in the:  1) femoral head, based on the proportion of bone involved (volume): score 0 = 0%, score 1 = 1–33%, score 2 = 34–66%, score 3 = 67–100%;  2) acetabulum: 0 = no, 1 = mild, 2 = moderate/significant; and  3) femoral neck (0 = no, 1 = yes). | | | | | | | | | | | | | | | |
| BMC (0–3) | | | Right femoral epiphysis | | | | | | | | Left femoral epiphysis | | | | |
|  |  |  |  | | | | | | | |  | | | | |
| BMC (0–2) | | | Right acetabulum | | | | | | | | Left acetabulum | | | | |
|  |  |  |  | | | | | | | |  | | | | |
| BMC (0–1) | | | Right femoral neck | | | | | | | | Left femoral neck | | | | |
|  |  |  |  | | | | | | | |  | | | | |
| **DESTRUCTIVE CHANGES: FLATTENING, EROSIONS, CYSTS**  Based on 3-D spin-echo (SE) T1-W images and fluid-sensitive and post-contrast images when appropriate:  Flattening = a flattened femoral head as seen in the coronal plane (mid-section) compared to what is expected for age  Erosion = a bony depression in at least 2 planes  Active erosion = an erosion filled with enhancing pannus  Bone cysts = sharply delineated, enhancing lesions with high signal on fluid-sensitive sequences | | | | | | | | | | | | | | | |
|  | | | A. Flattening of the femoral head is first assessed subjectively at a 0–4 scale, and thereafter using a Mose circle, in increments of 25%: 0 = <10%, 1 = 10–25%, 2 = 26–50%, 3 = 51–75%, 4 = 76–100% | | | | | | | | | | | |  |
| Flattening  (0–4) | | |  | | | | Right femoral head | | | | | Left femoral head | | |  |
|  |  |  | Subjectively | | | |  | | | | |  | | |  |
|  |  |  | Mose circle | | | |  | | | | |  | | |  |
|  | | | | | | | | | | | | | | |  |
|  | | | B.1 Erosion. Assess the femoral head for bony erosions based on the proportion of femoral head volume involved, in increments of 25%: 0 = 0%, 1 = 1–25%, 2 = 26–50%, 3 = 51–75%, 4 = 76–100% | | | | | | | | | | | |  |
| Erosion  (0–4) | | | Right femoral head volume | | | | | | | | Left femoral head volume | | | |  |
|  |  |  |  | | | | | | | |  | | | |  |
|  | | | Right femoral head | | | | | | | | Left femoral head | | | |  |
| Active erosion (no/yes = 0/1) | | |  | | | | | | | |  | | | |  |
|  | | | Right femoral head | | | | | | | | Left femoral head | | | |  |
| Bone cysts (no/yes = 0/1) | | |  | | | | | | | |  | | | |  |
|  | | | | | | | | | | | | | | |  |
|  | | | B.2 Assess the femoral neck for bony erosions (0=no, 1=yes) and cyst. Small irregularities at the physis are be scored as 0 | | | | | | | | | | | |  |
|  | | | Right femoral neck | | | | | | | | Left femoral neck | | | |  |
| Erosion  (0–1) | | |  | | | | | | | |  | | | |  |
| Bone cysts  (no/yes = 0/1) | | |  | | | | | | | |  | | | |  |
|  | | | B.3 Assess the acetabulum for bony erosions, based on the surface of involvement of the acetabular margin, as 0 = no erosion, 1 = 1–33% of margin eroded, 2 = 34–66% of margin eroded, 3 = 67–100% of margin eroded.  Simplified scoring for acetabulum: 0 = no irregularities, 1 = bone irregularities, 2 = bone erosions (0/2) | | | | | | | | | | | |  |
|  | | | Right acetabulum | | | | | | | | | Left acetabulum | | |  |
| Erosion  (0–3) | | |  | | | | | | | | |  | | |  |
| Simplified  erosion (0–2) | | |  | | | | | | | | |  | | |  |
| Bone cysts (no/yes = 0/1) | | |  | | | | | | | | |  | | |  |
| **CARTILAGE DAMAGE**  Measure the **joint cartilage thickness** (mm) on post-contrast 3-D GRE T1-W sequences superiorly (mid-weight-bearing area) and next, score subjectively as normal, mildly, moderately or severely narrowed (0–3 scale).  Assess the cartilage in terms of **signal abnormalities** and morphological changes scored in a 0–4 scale: 0 = no cartilage changes, 1 = signal abnormalities without morphological defects, 2 = morphological changes involving 0–33% of the joint surface, 3 = morphological changes involving 34–66% of the joint surface, 4 = morphological changes involving 67–100% of the joint surface.  Assess **symmetry**: right vs. left joint space height (JSH): 0 = symmetrical, 1 = right<left, 2 = right>left | | | | | | | | | | | | | | | |
|  | | | | | Right hip | | | | | | | Left hip | | | |
| Joint space width (mm) | | | | |  | | | | | | |  | | | |
| Narrowed JSH (0–3) (no–mild–moderate–severe) | | | | |  | | | | | | |  | | | |
| Cartilage changes (0–4) (signal/loss %) | | | | |  | | | | | | |  | | | |
| Symmetry (0–2) (only one score for both) | | | | |  | | | | | | |  | | | |
|  | | | | | | | | | | | | | | | |
| **POTENTIAL GROWTH ABNORMALITIES (assessed similarly to radiographs)**  **Based on coronal T1-W MRI sequences** | | | | | | | | | | | | | | | |
|  | | | | | | | | | | Right hip | | | Left hip | | |
| Femoral neck width (in mm, outer cortex) | | | | | | | | | |  | | |  | | |
| Femoral head/neck length (in mm, outer cortex) | | | | | | | | | |  | | |  | | |
| CCD-angle | | | | | | | | | |  | | |  | | |
| Troch–fem head distance (outer cortex) | | | | | | | | | |  | | |  | | |
| Closed physis (0=no, 1=yes) | | | | | | | | | |  | | |  | | |
| Coxa magna (0=no, 1=yes) | | | | | | | | | |  | | |  | | |
| Coxa brevis (0=no, 1=yes) | | | | | | | | | |  | | |  | | |
| Protrusio acetabulae (0=no, 1=yes) | | | | | | | | | |  | | |  | | |
| Fovea enlargement (0=no, 1=probably, 2=definitely) | | | | | | | | | |  | | |  | | |
|  | | | | | | | | | | | | | | | |
| **SECONDARY POST-INFLAMMATORY CHANGES**  **Based on both TSE T1 and fluid-sensitive FS sequences** | | | | | | | | | | | | | | | |
|  | | | | | | | | | | Right hip | | | Left hip | | |
| Osteophytes (0=no, 1=yes) | | | | | | | | | |  | | |  | | |
| Sclerotization (0=no, 1=yes) | | | | | | | | | |  | | |  | | |
| **Comments:** | | | | | | | | | | | | | | | |

*3-D* three-dimensional, *FS* fat saturated, *GRE* gradient echo, *JIA* juvenile idiopathic arthritis, *Lt* left, *OPBG* Bambino Gesù Children’s Hospital, *Rt* right, *SI* signal intensity, *STIR* short tau inversion recovery, *T1-W* T1-weighted, *T2-W* T2-weighted

AROME 30.10.16, adjusted in Rome as of 18.11.16, in Amsterdam as of 22.10.17 and in Rome as of 15.01.18
