# Supplementary material for: A novel magnetic resonance imaging scoring system for active and chronic changes in children and adolescents with juvenile idiopathic arthritis of the hip
Source: Pediatr Radiol. 2022 Sep 23;53(3):426–37. doi: 10.1007/s00247-022-05502-8 (PMC9968695; doi:10.1007/s00247-022-05502-8)
Supplement: Supplementary file 2 — Supplementary file2 (PDF 13535 KB) Online Supplementary Material 2 Imaging atlas with relevant examples of each variable and grade in juvenile idiopathic arthritis (JIA) [file 247_2022_5502_MOESM2_ESM.pdf]

Online Supplementary Material 2 : Hip MRI Scoring Atlas

MRI Scoring Atlas

Juvenile Idiopathic Arthritis of the Hips

A Guide to Application of a Novel Scoring System

General Scoring Principles:

1. Score predominant finding
2. If in doubt, be conservative

ENHANCEMENT INTENSITY

0 = NONE/ SUBTLE, 1 = MILD, 2 = MODERATE (SI < VESSELS), 3 = SEVERE (SI > VESSELS)

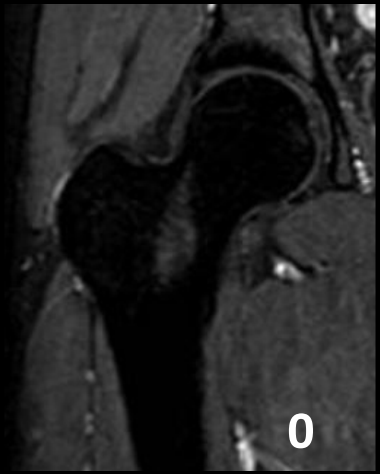

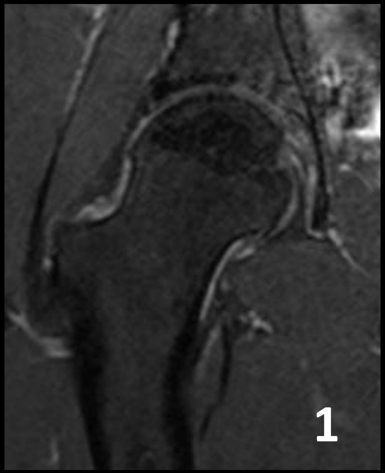

Score 1+ (the lateral aspect [yellow arrow] was predominant finding when scrolling)

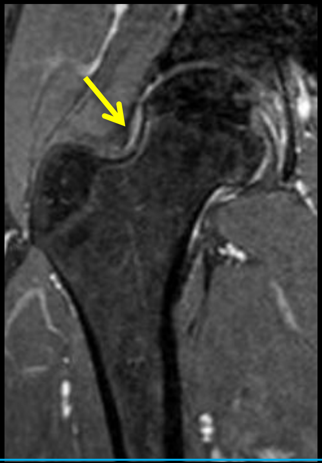

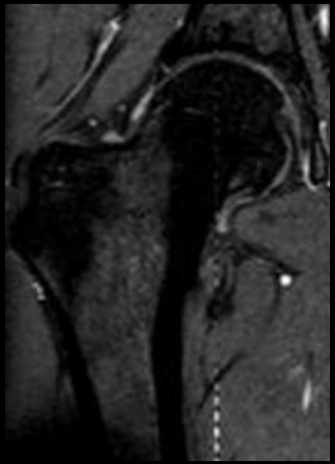

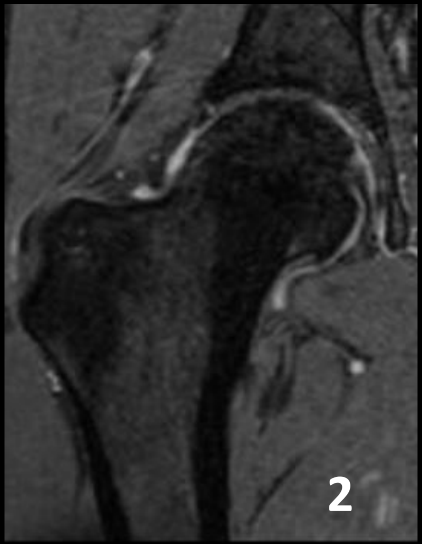

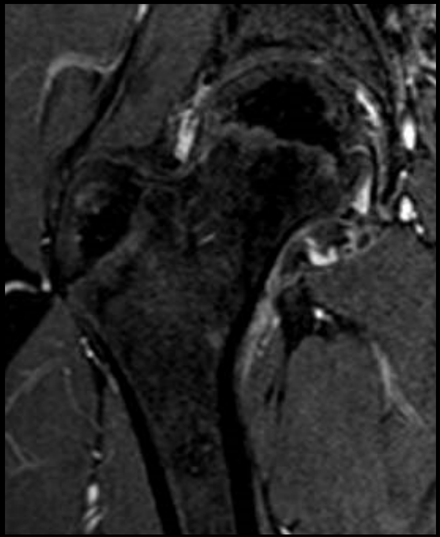

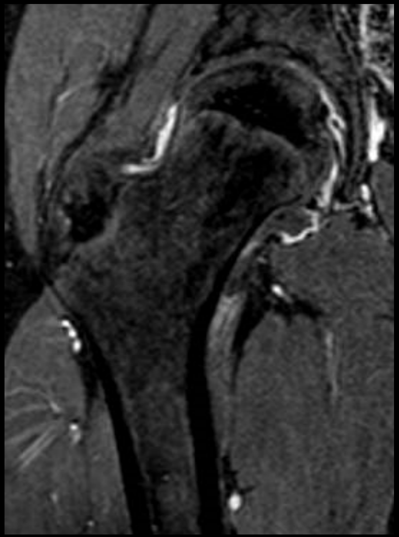

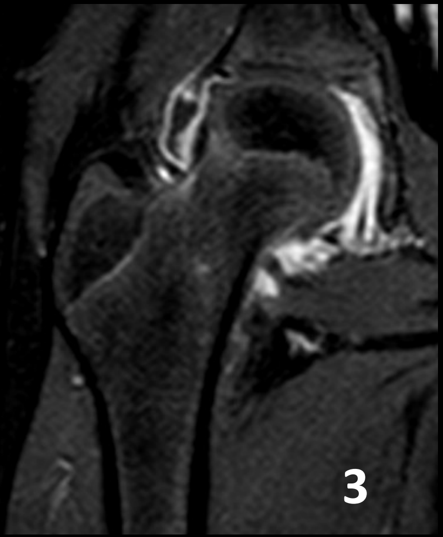

SYNOVIAL THICKENING (SUBJECTIVE SCORING – T1FS + Gd)

0 = NOTHING, 1 = MILD, 2 = MODERATE, 3 = SEVERE

0

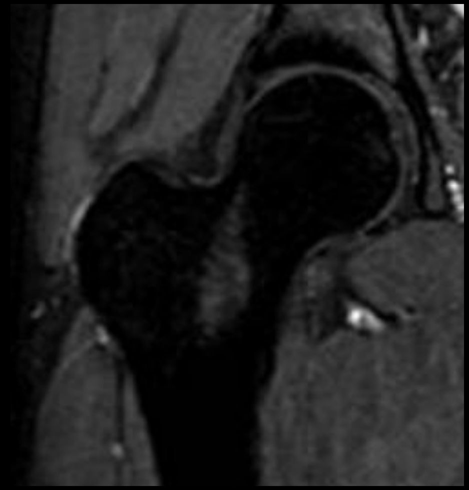

1

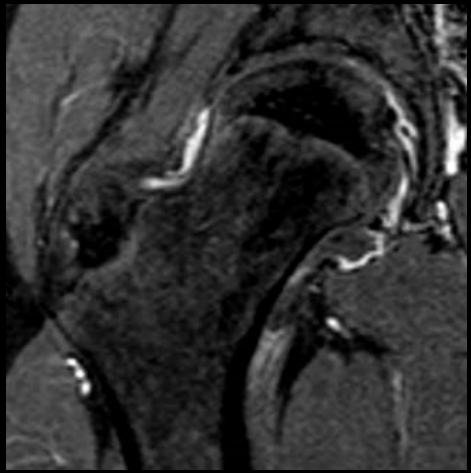

2

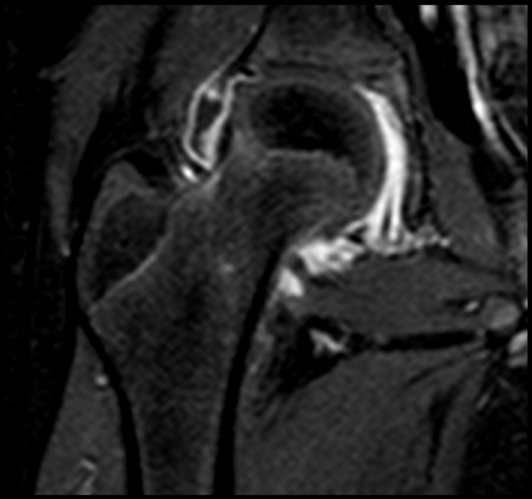

3

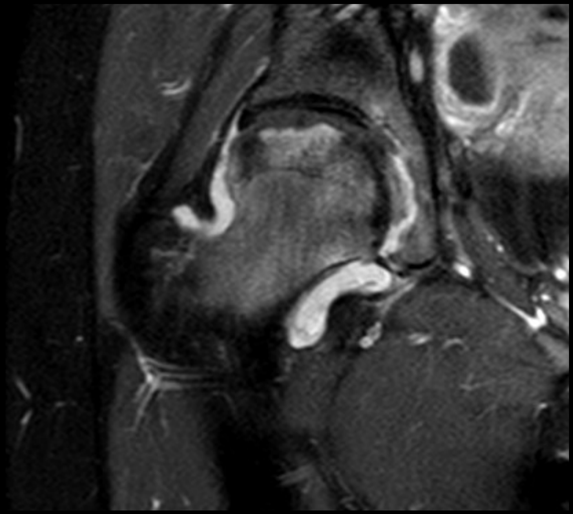

SYNOVIAL THICKENING

(MEDIAL AND LATERAL – T1FS + Gd)

FIND A MID SECTION, APPROX WHERE YOU SEE FOVEA

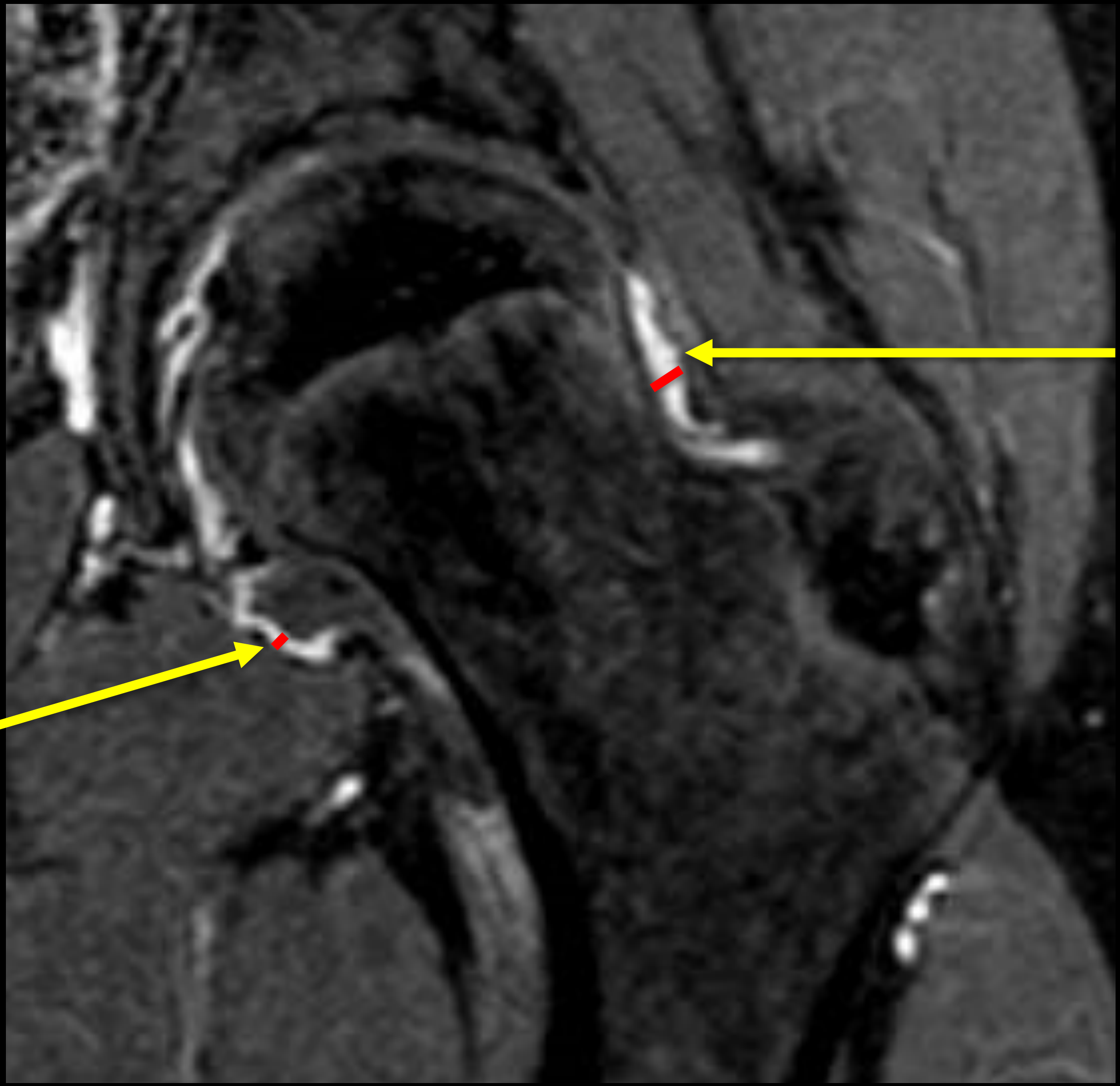

Lateral– here you must divide measurement by 2 (i.e. half the measurement

Medial – as you can see one layer this measure is ok

SYNOVIAL THICKENING
(AXIAL – T1FS + Gd)

FIND A MID SECTION, NOT TOO SUPERIOR OR INFERIOR

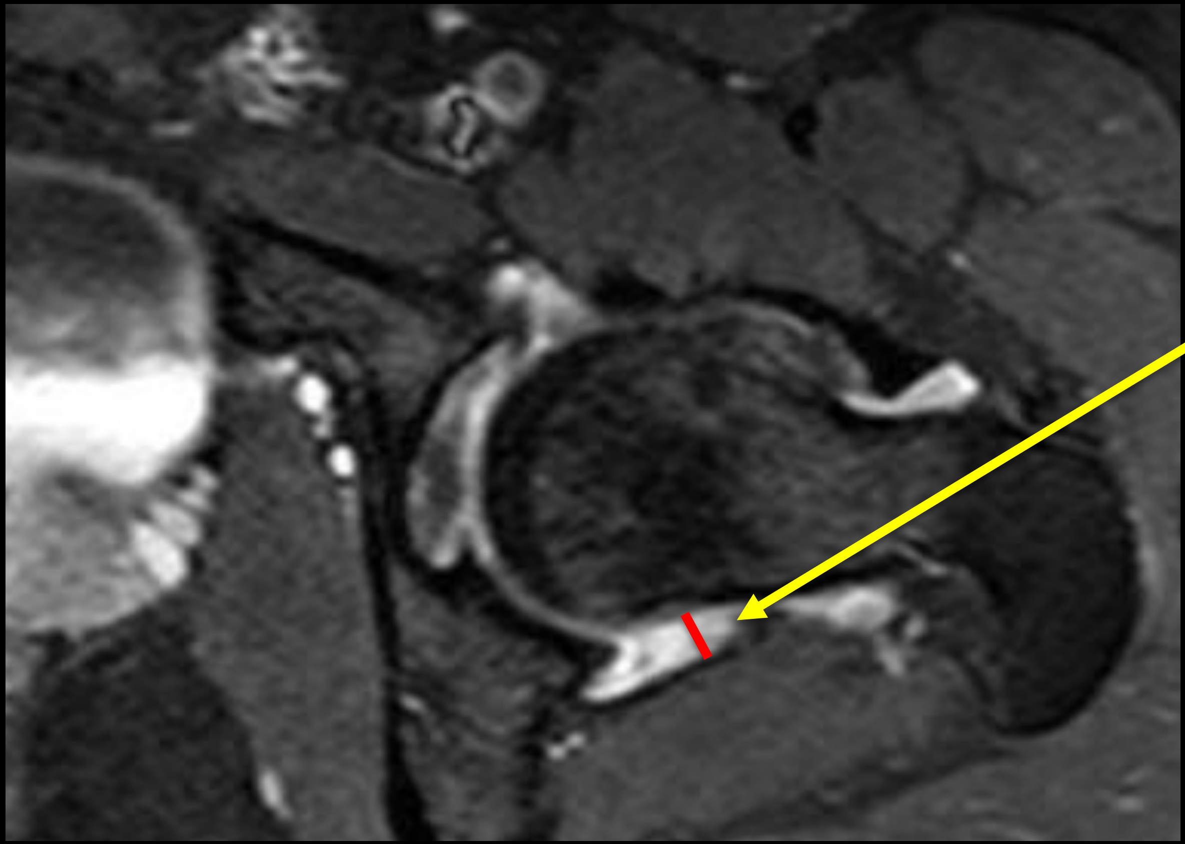

MEASURE ANTERIOR OR POSTERIOR – UP TO YOU.

Remember to divide by 2 if you are measuring 2 layers, as in this example

OVERALL SYNOVIAL INFLAMMATION (ENHANCEMENT + THICKENING – T1+C)

0 = NONE/ SUBTLE, 1 = MILD, 2 = MODERATE, 3 = SEVERE

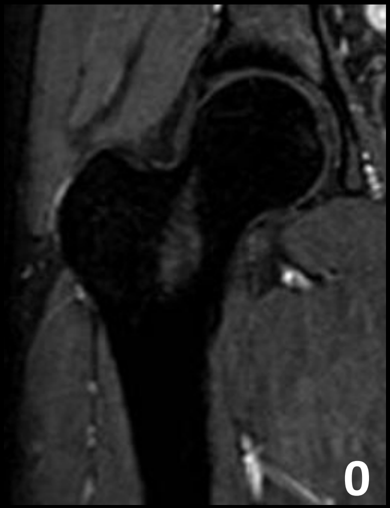

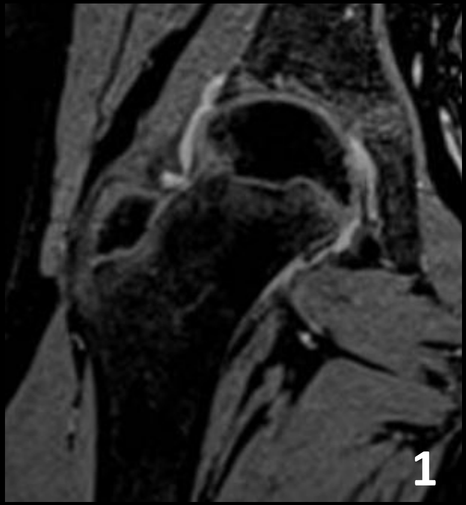

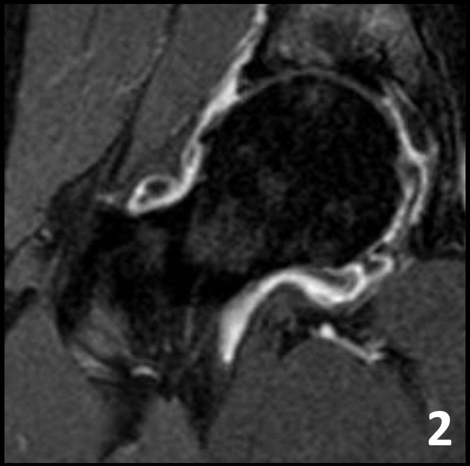

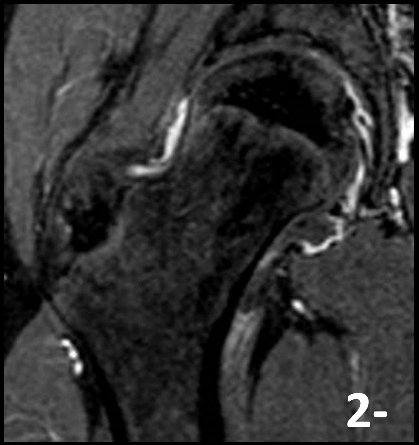

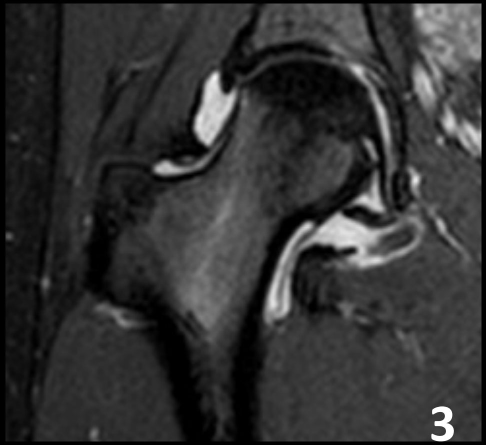

EFFUSION (T2FS)

(0 = NONE), 1 = SLIVER, 2 = MILD, 3 = MODERATE, 4 = LARGE AMOUNT

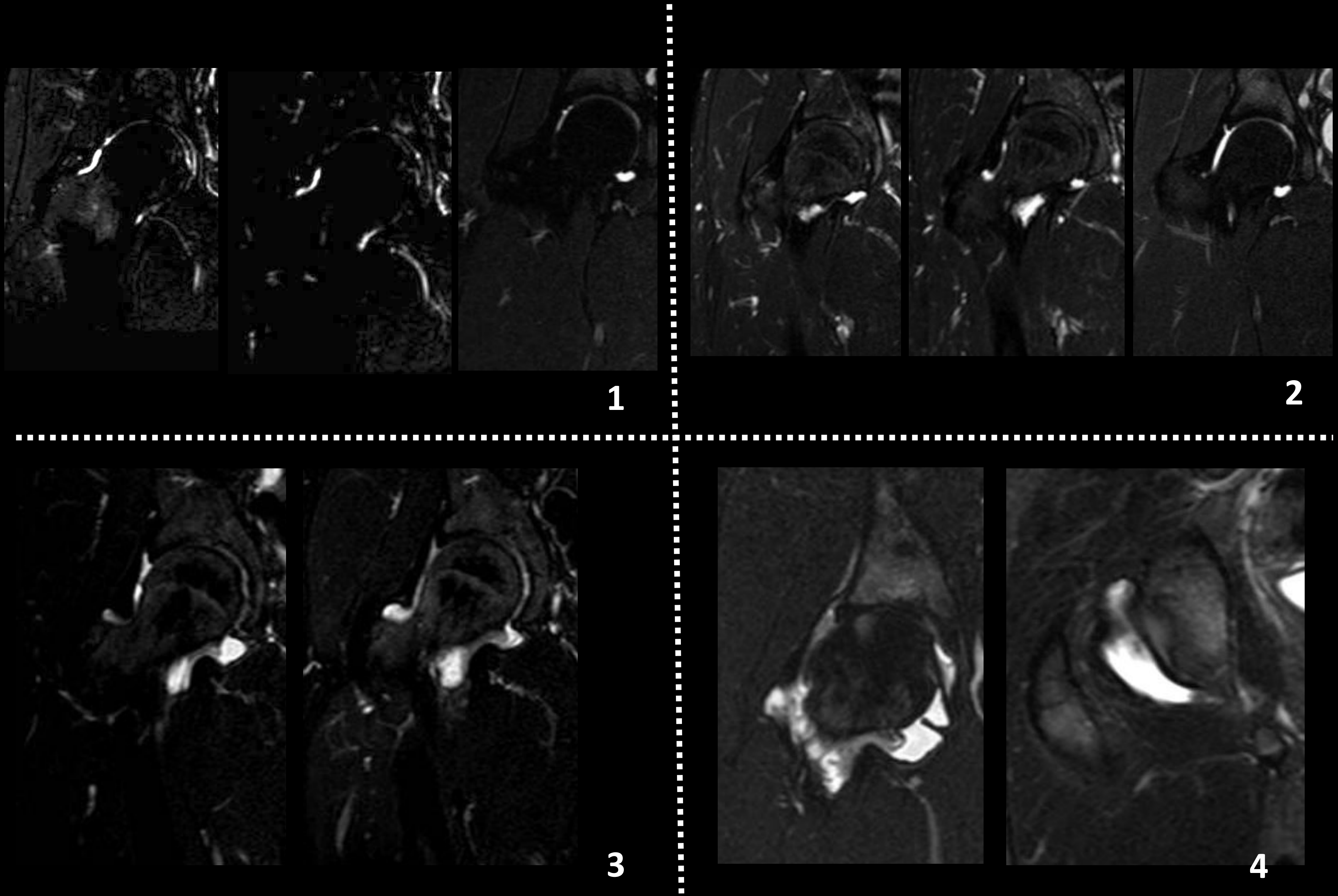

OVERALL INFLAMMATION inc EFFUSION (T1FS + Gad / T2FS)

1 = MILD

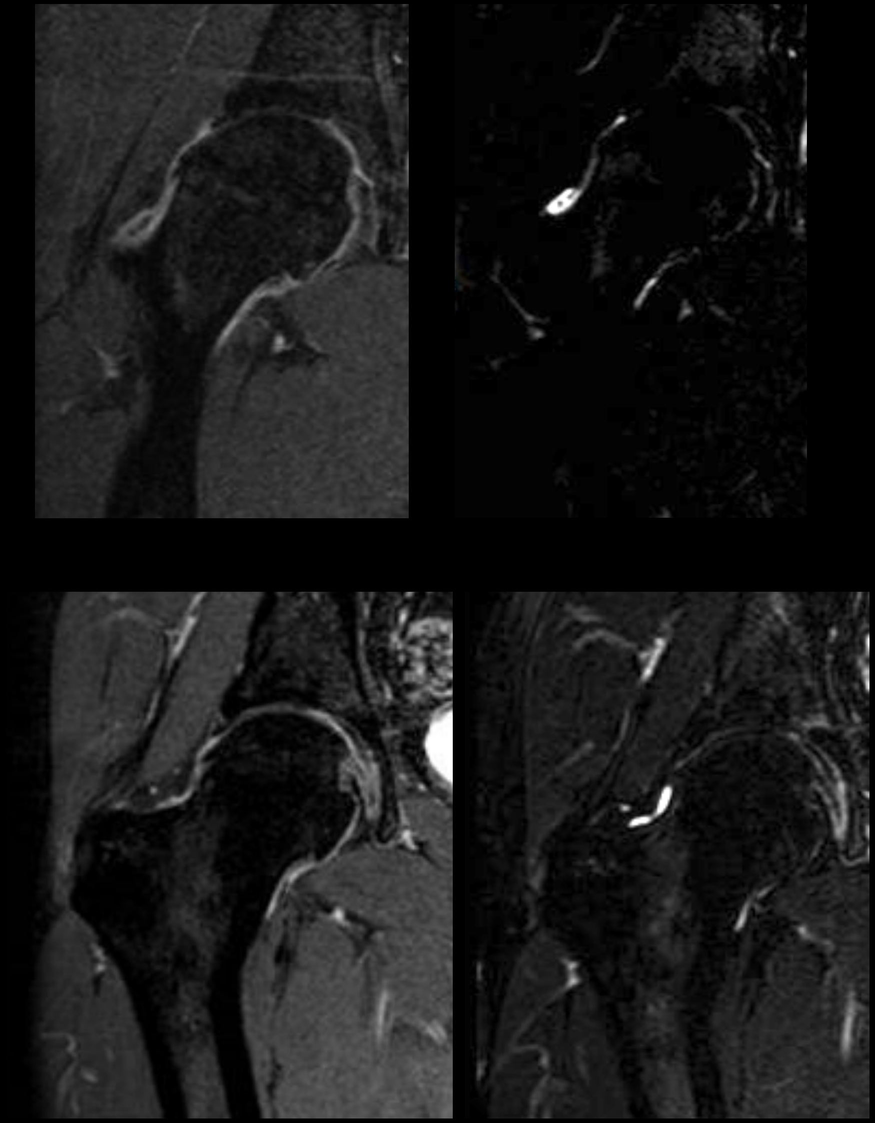

0 = NONE

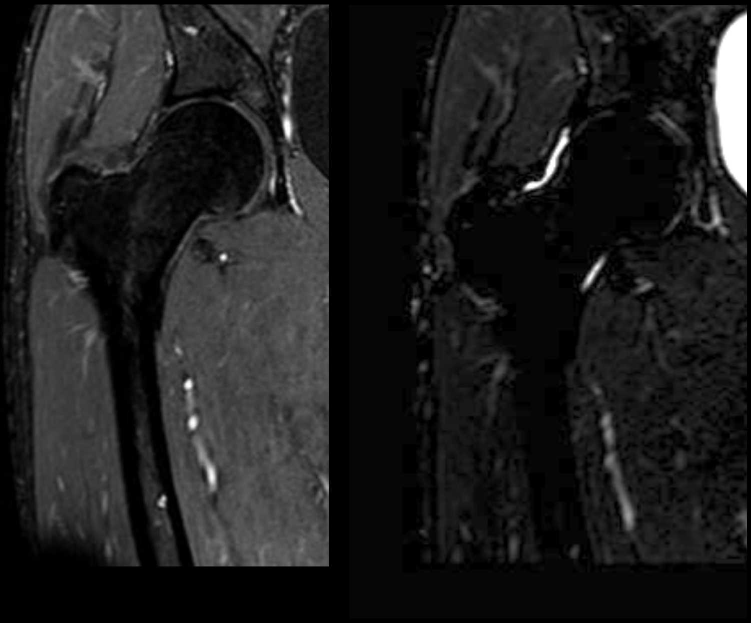

OVERALL INFLAMMATION inc EFFUSION (T1FS + Gad / T2FS)

2 = MODERATE

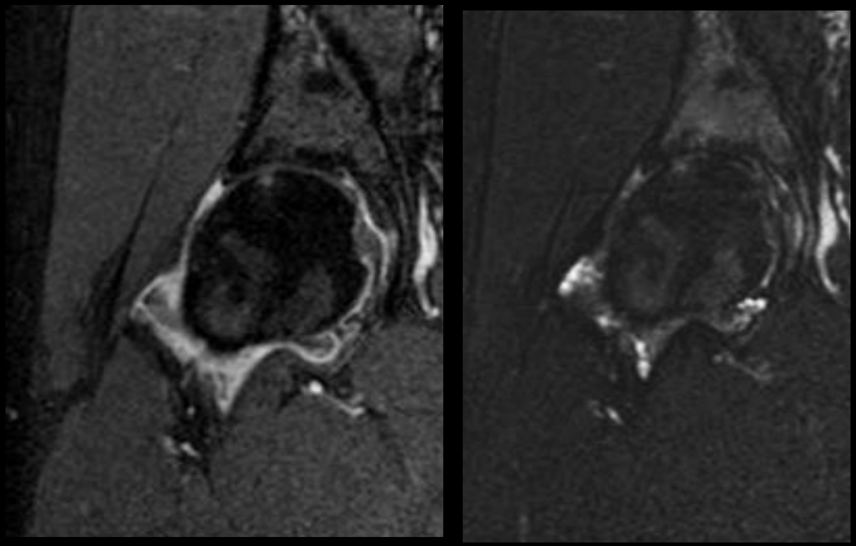

3 = SEVERE

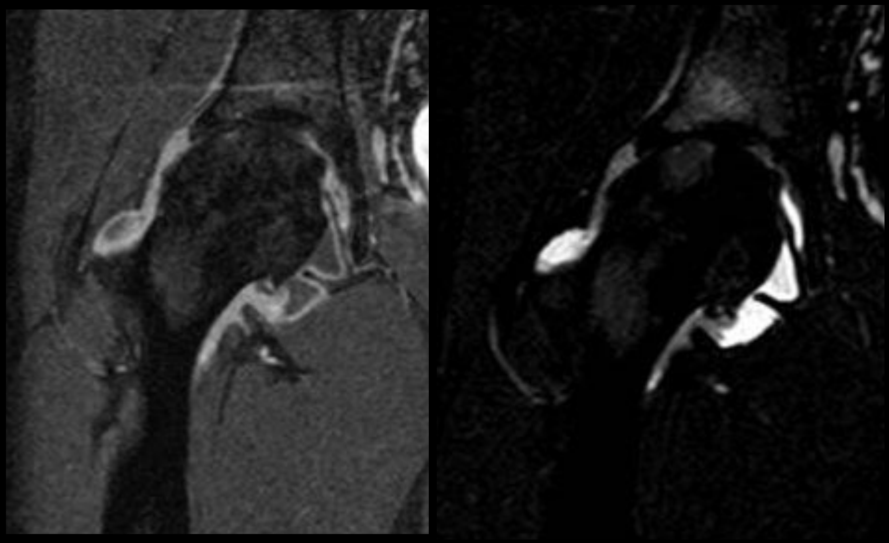

BONE MARROW CHANGES
FEMORAL HEAD

0=0%
1=1-33%
2= 34-66%,
3= 67%-100%

SCORE 1 – (T1 + T1C)

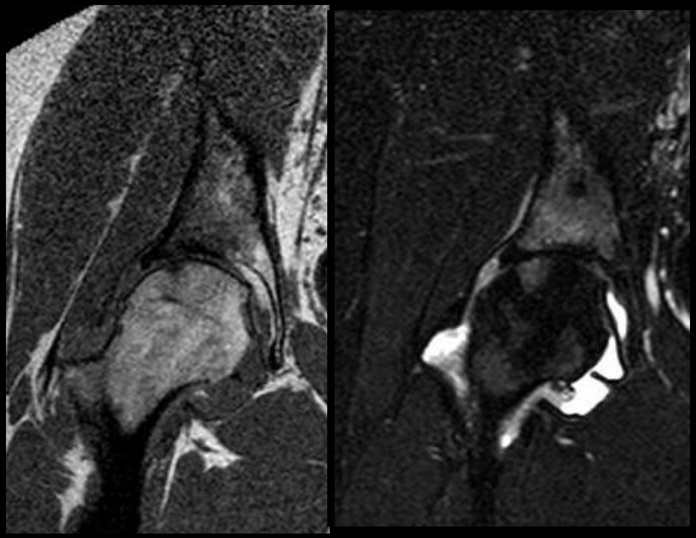

SCORE 2 – (T1 + T1C)

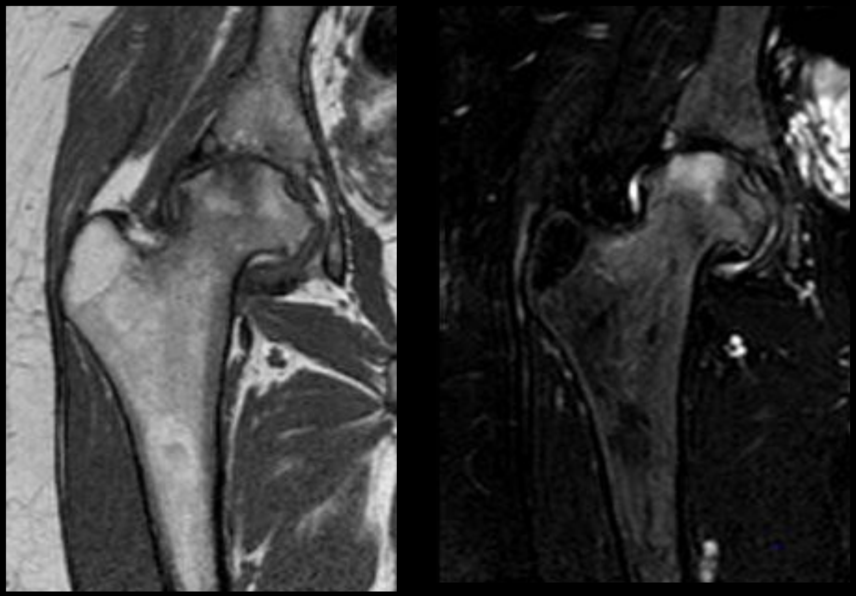

SCORE 3 – (T1 + T1C)

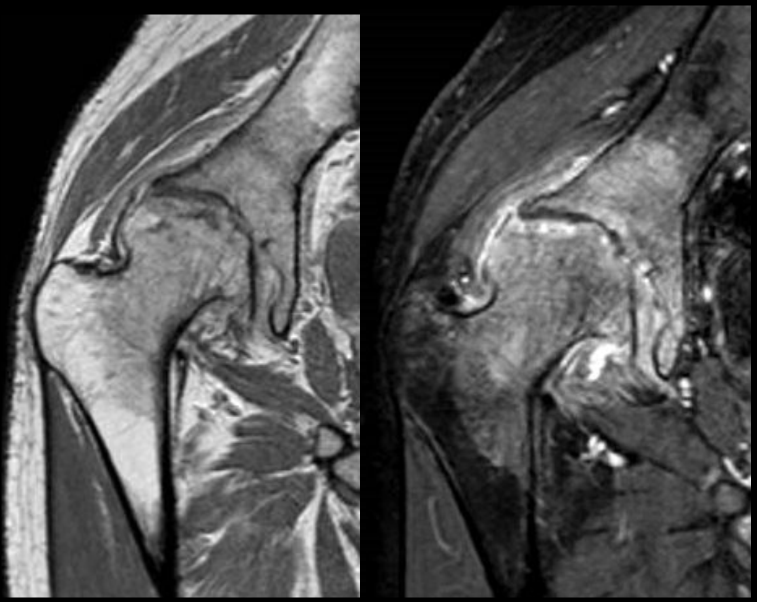

SCORE 1 – (T1 + T2FS)

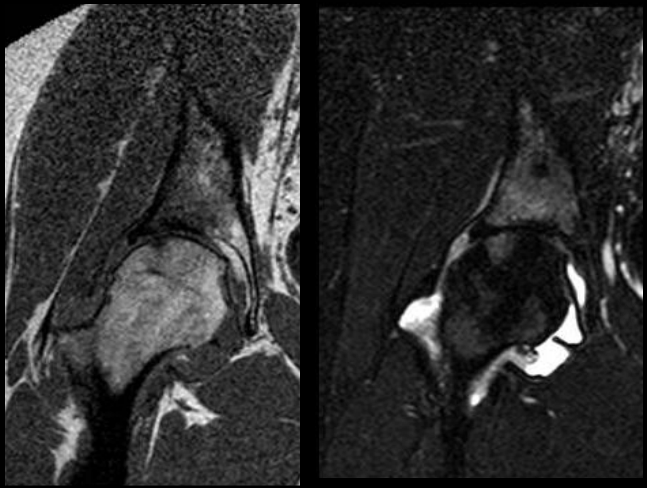

SCORE 1 – (T1 + T2FS)

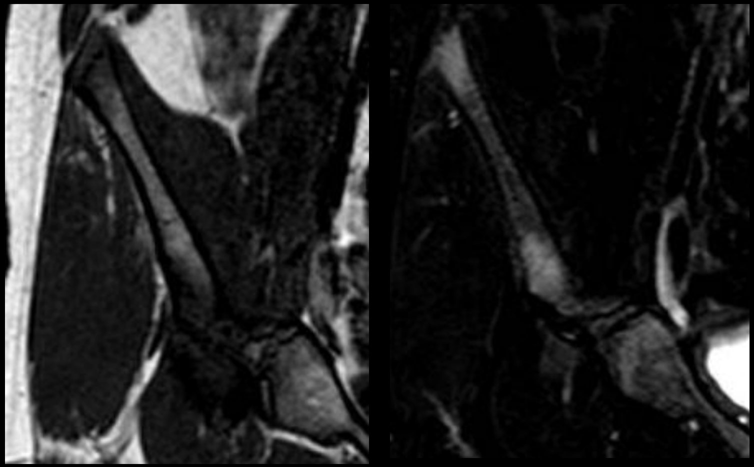

BONE MARROW CHANGES ACETABULUM (Joint surface + 2cm rim)

0= NONE
1= MILD
2= MODERATE /SIGNIFICANT

SCORE 2 – (T1 + T2FS)

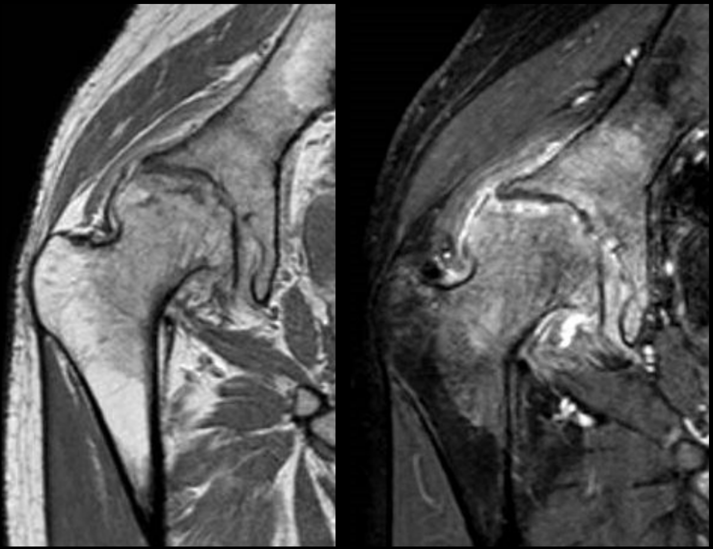

1= PRESENT

BONE MARROW CHANGES
FEMORAL NECK

0= NONE
1= PRESENT

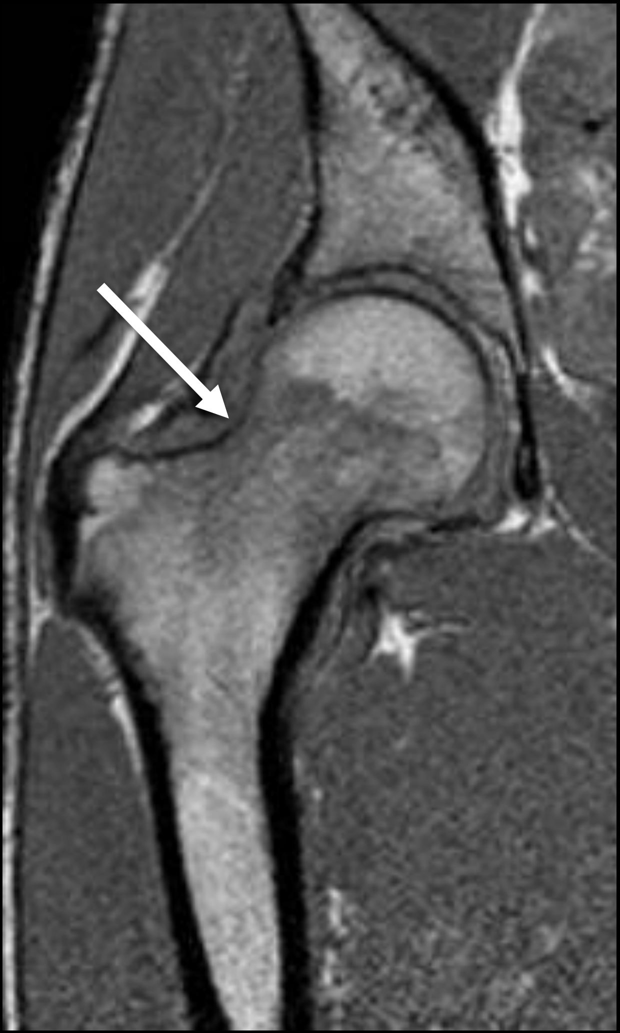

T1

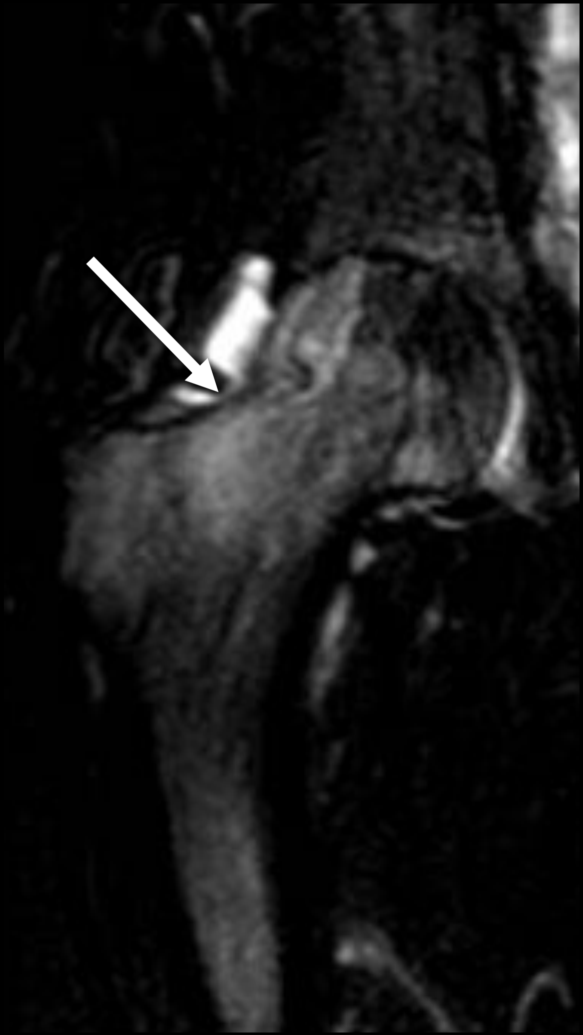

T2 + FS

EROSIONS OF FEMORAL HEAD :

0= 0%, 1= 1-25%, 2= 26-50%, 3 = 51 -75%, 4 = 76 - 100%

SCORE 1 (T1, T1c)

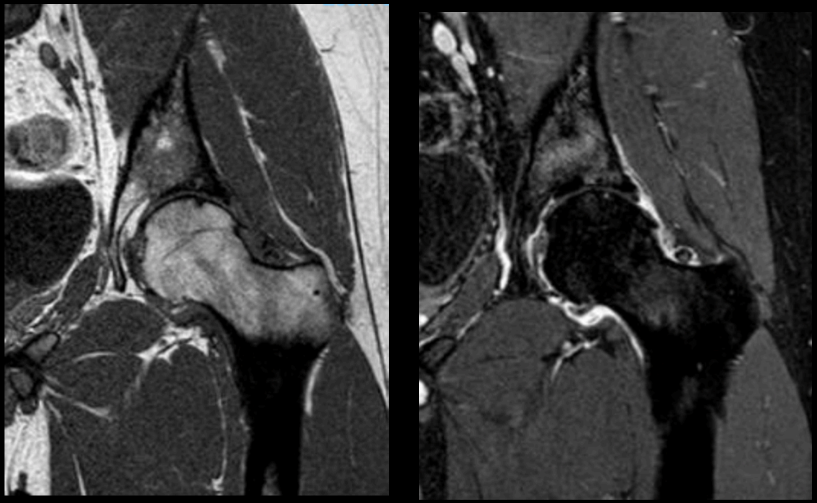

SCORE 2 (T1, T1c)

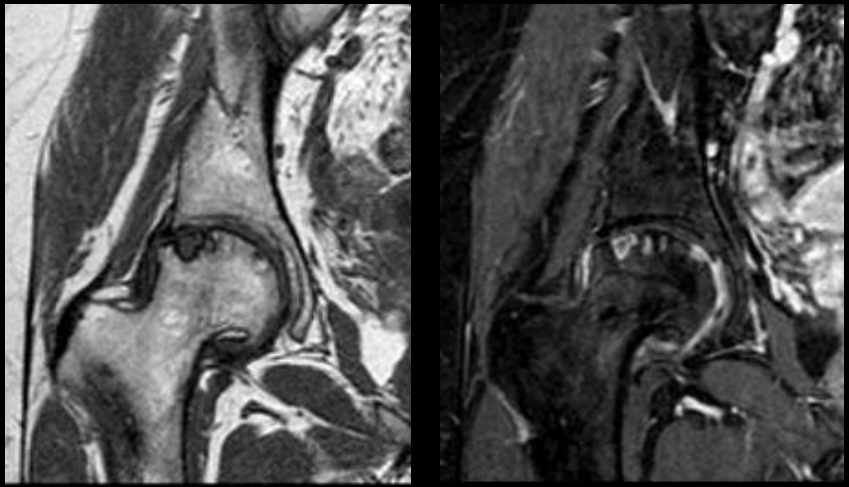

SCORE 3 (T1, T1c)

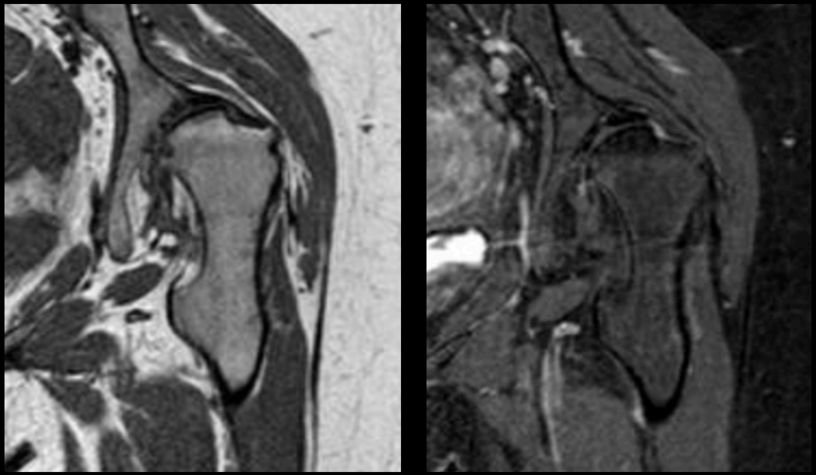

SCORE 4 (T1, T1c)

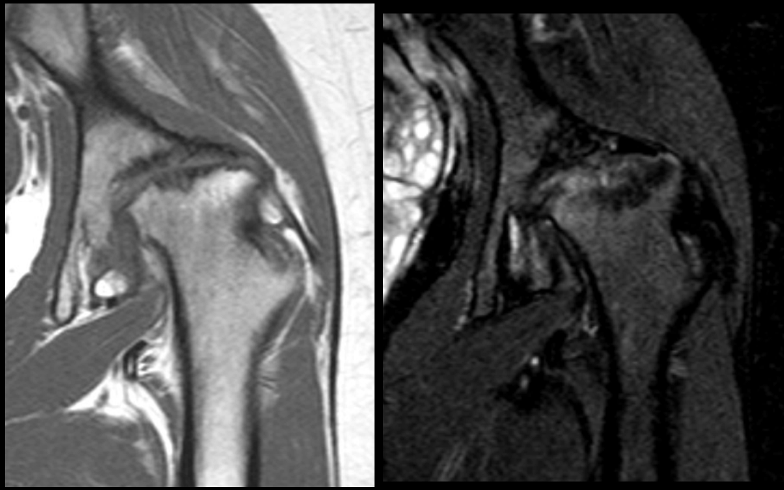

EROSIONS OF ACETABULUM: 0= 0%, 1= 1- 33%, 2= 34- 66%, 3 = 67 -100%

SCORE 0 (T1, T1c)

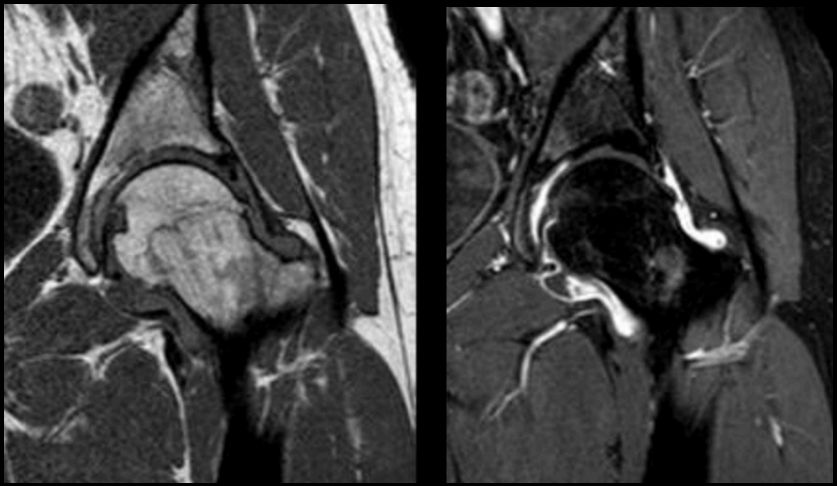

SCORE 1 (T1, T1c)

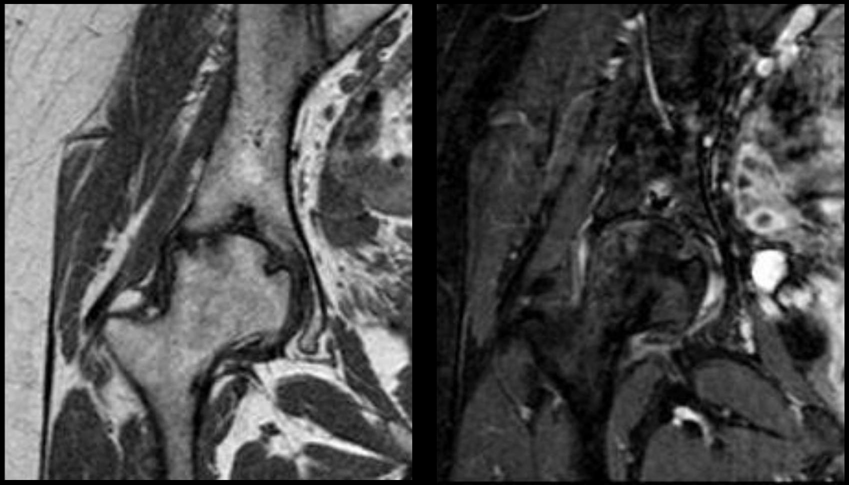

SCORE 2 (T1, T1c)

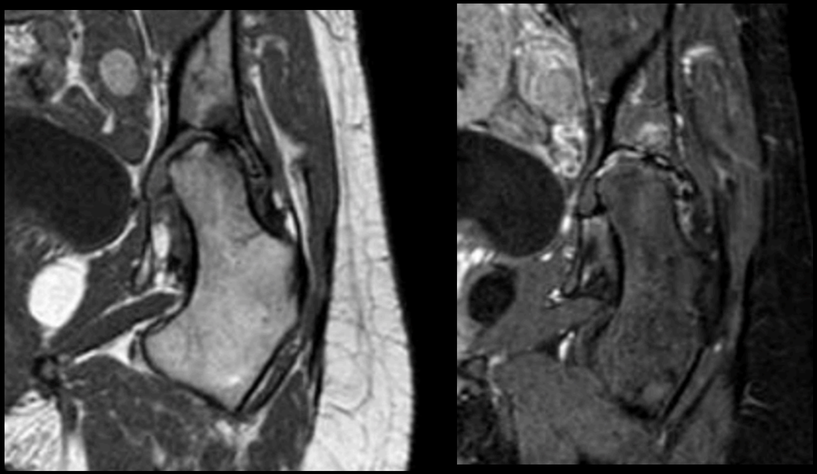

SCORE 3 (T1, T1c)

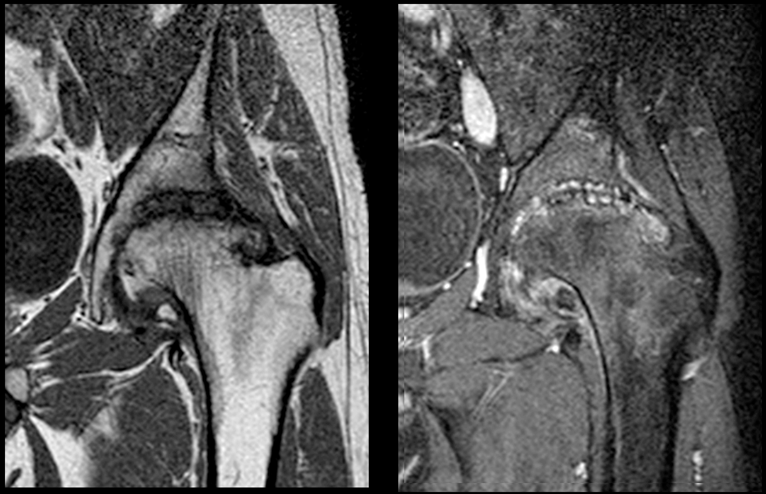

EROSIONS OF ACETABULUM (SIMPLIFIED)

0= NONE
1= BONY IRREGULARITIES
2= BONE EROSION

SCORE 0 – (T1, T1c)

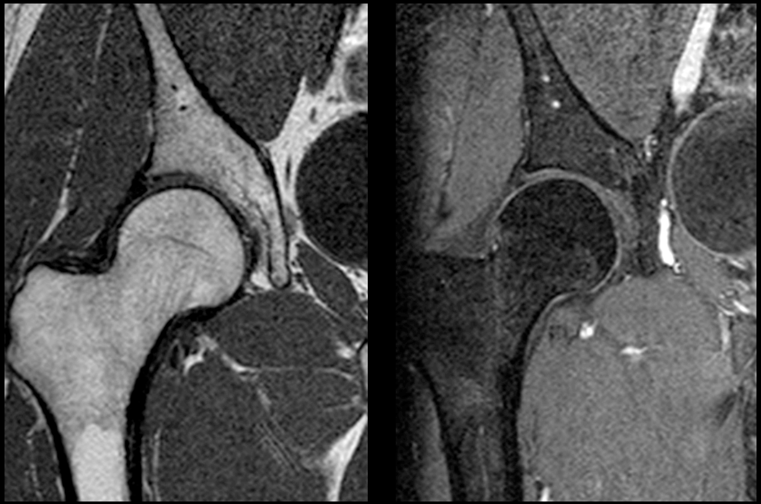

SCORE 1 – (T1, T1c)

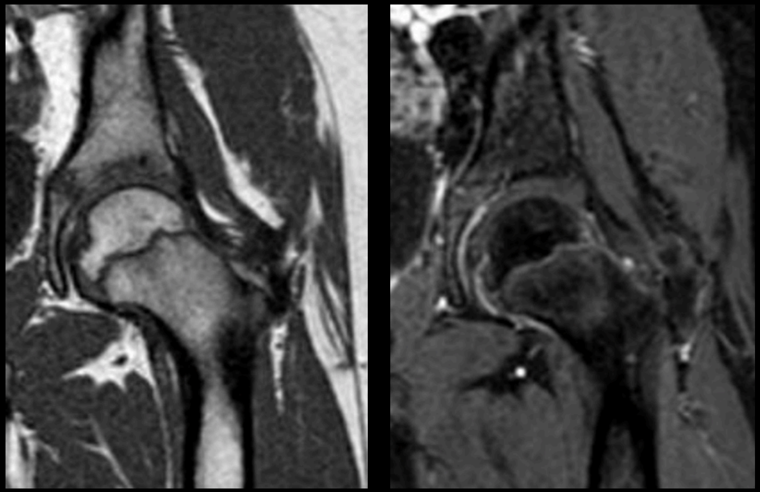

SCORE 2 – (T1, T1c)

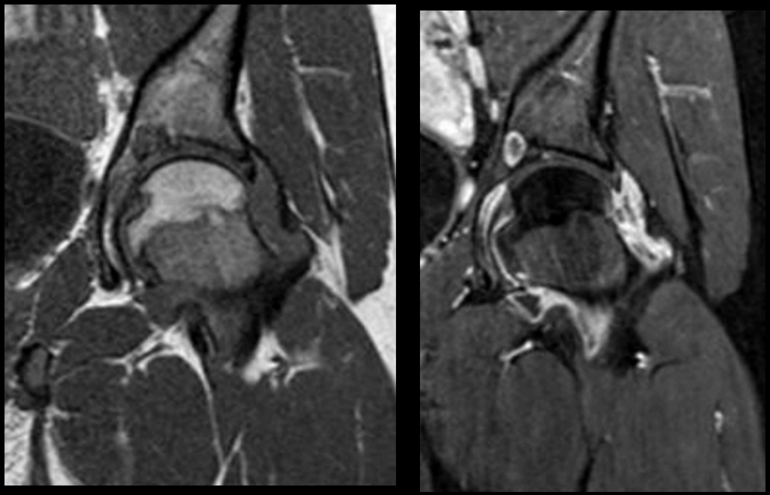

EXAMPLE OF BONE CYST : 0 = NONE, 1 = PRESENT

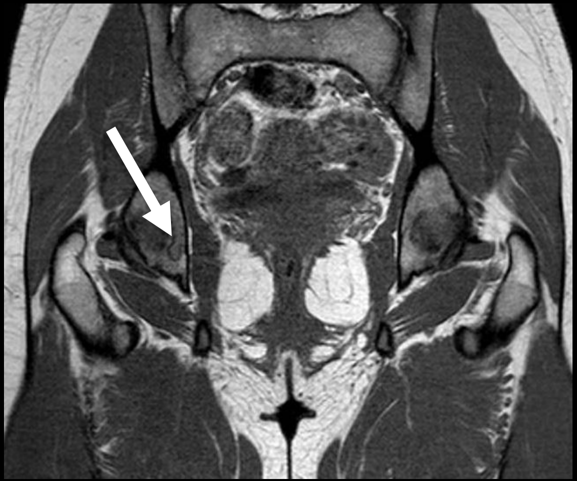
T1

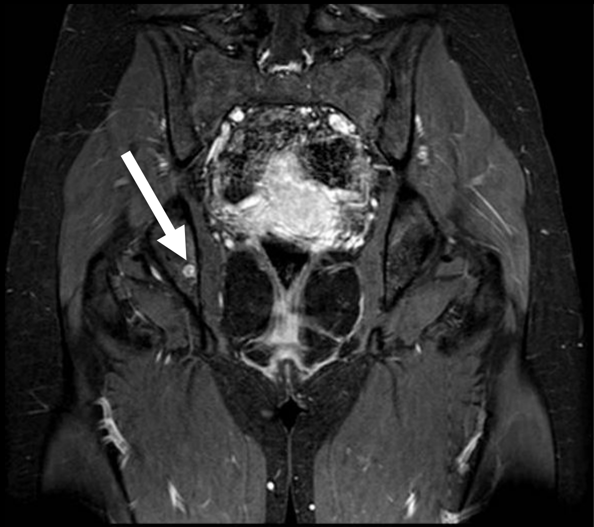
T1 FS + CONTRAST

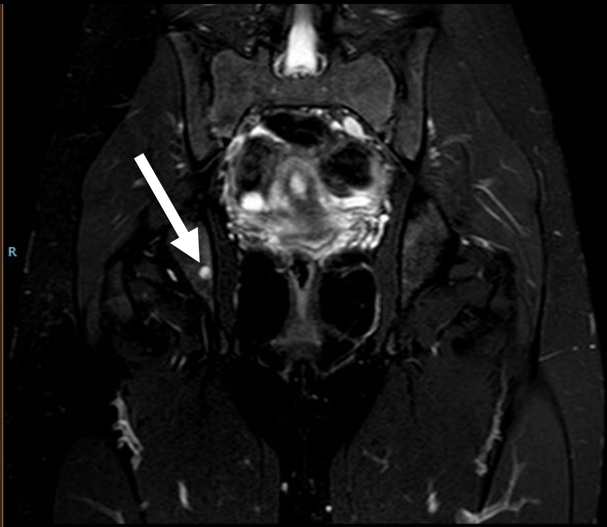
T2FS

EXAMPLE OF ACTIVE EROSION

0 = NONE
1 = PRESENT

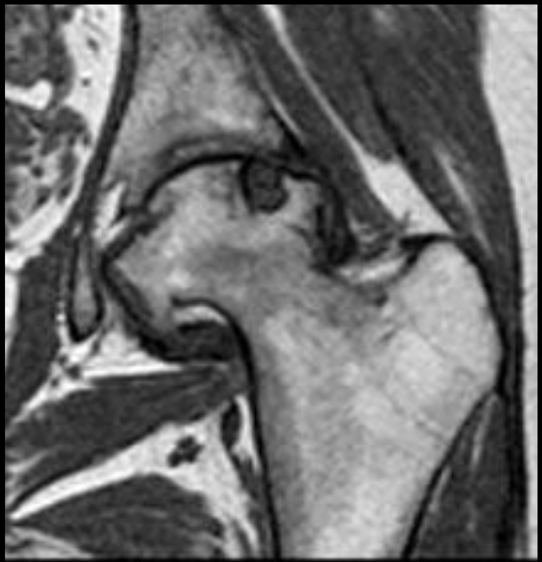
T1

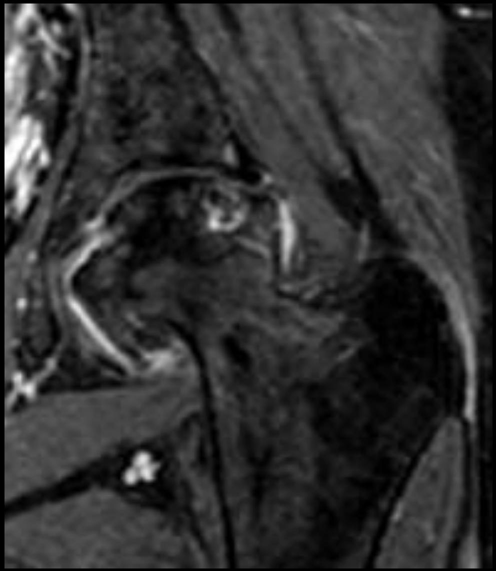
T1 FS + CONTRAST

EXAMPLE OF OSTEOPHYTES

0 = NONE
1 = PRESENT

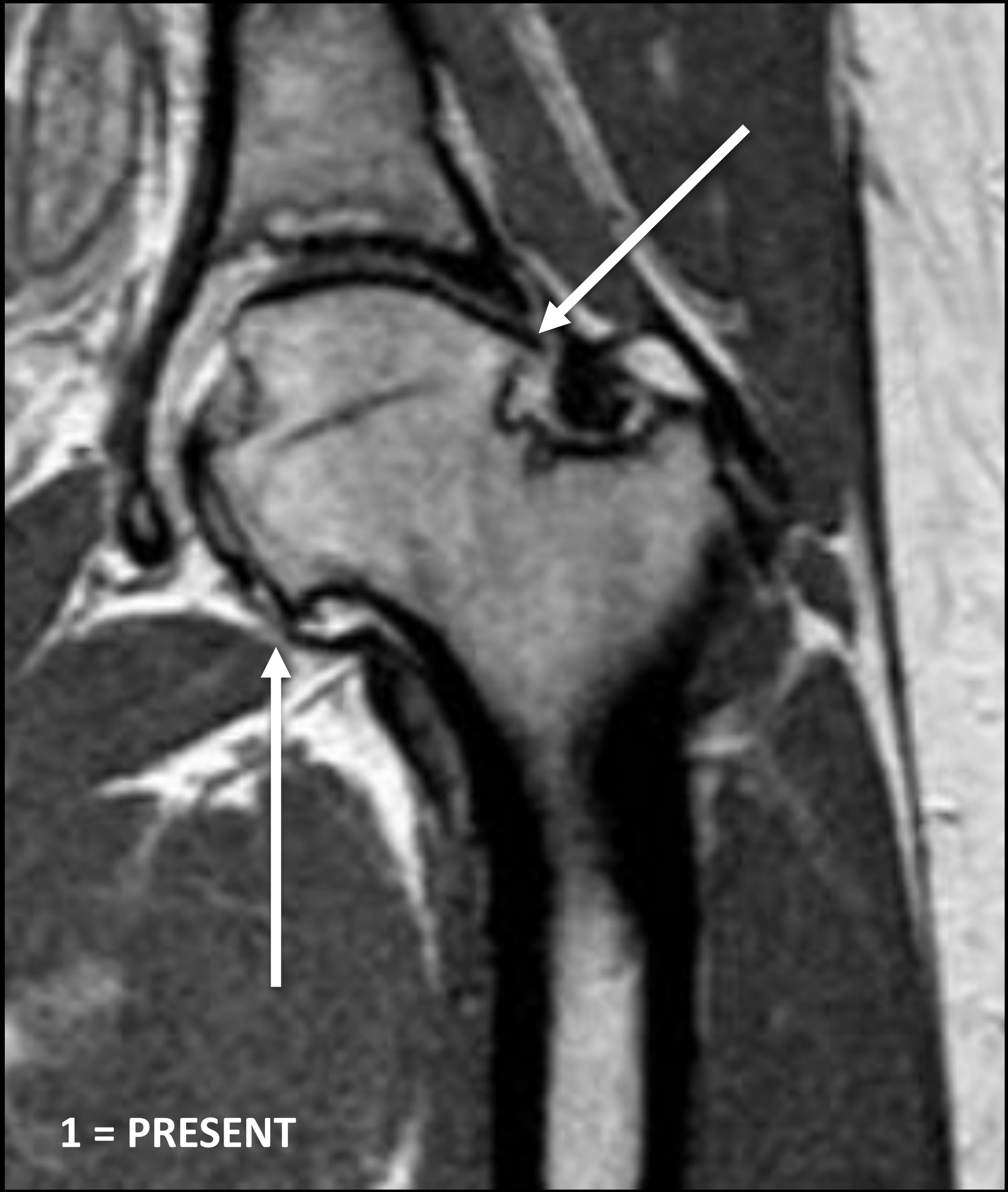

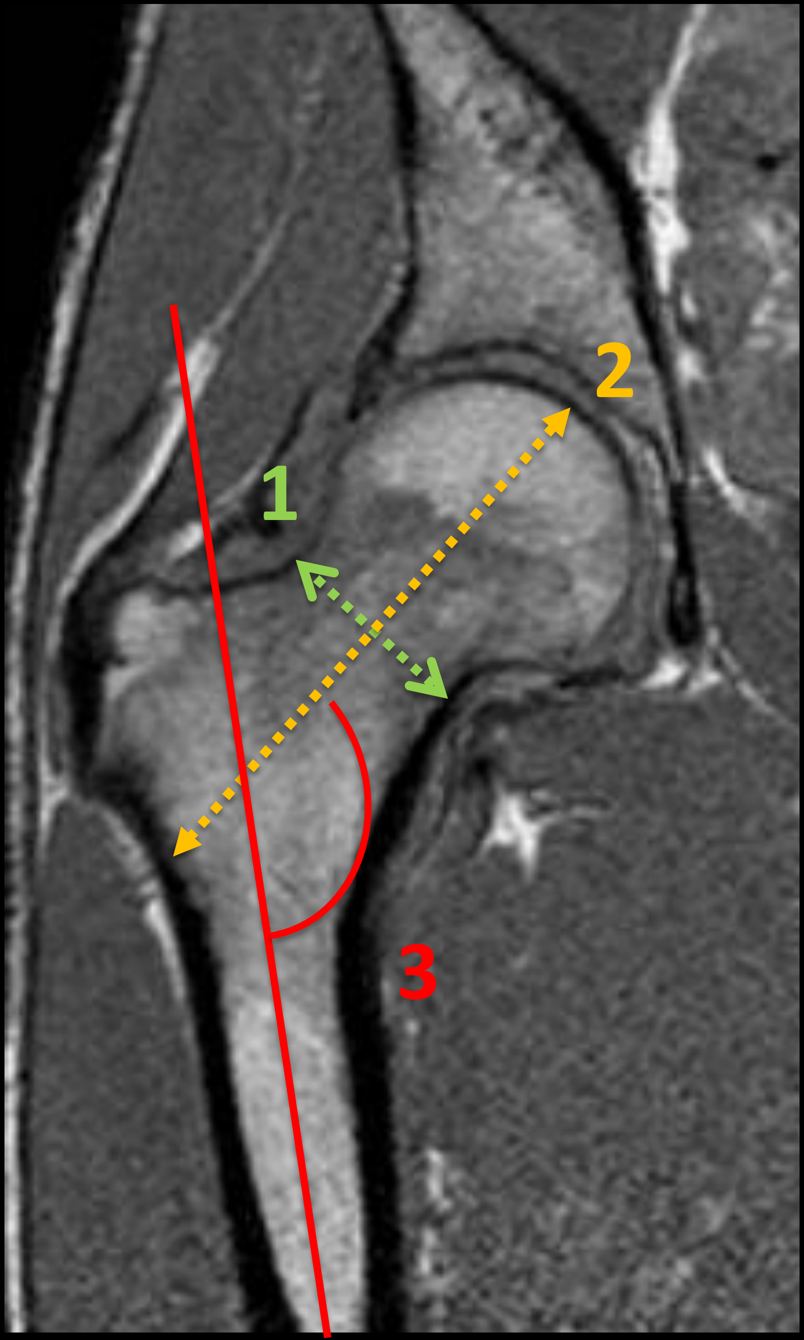

For all measurements, perform as per X-ray hip scoring paper, but pick a mid-section T1 image.

1. Draw a width of femoral neck (approx mid-neck)

2. At 90 degrees to this line, draw the femoral neck length

3. Draw a line through the femoral shaft. Where this line meets the femoral neck length line, measure the CCD angle.

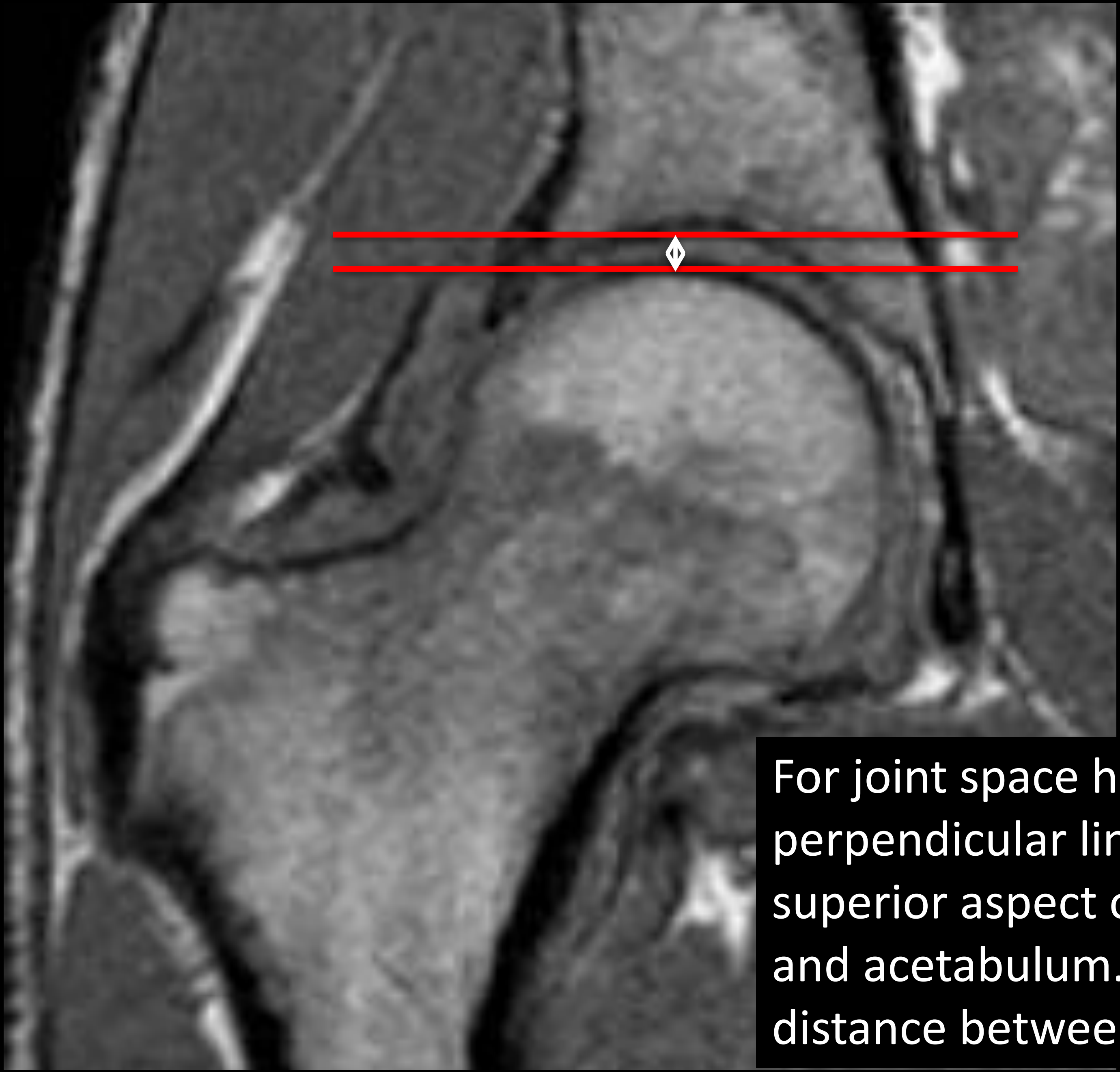

For joint space height, draw two perpendicular lines along superior aspect of femoral head and acetabulum. Measure distance between these lines.

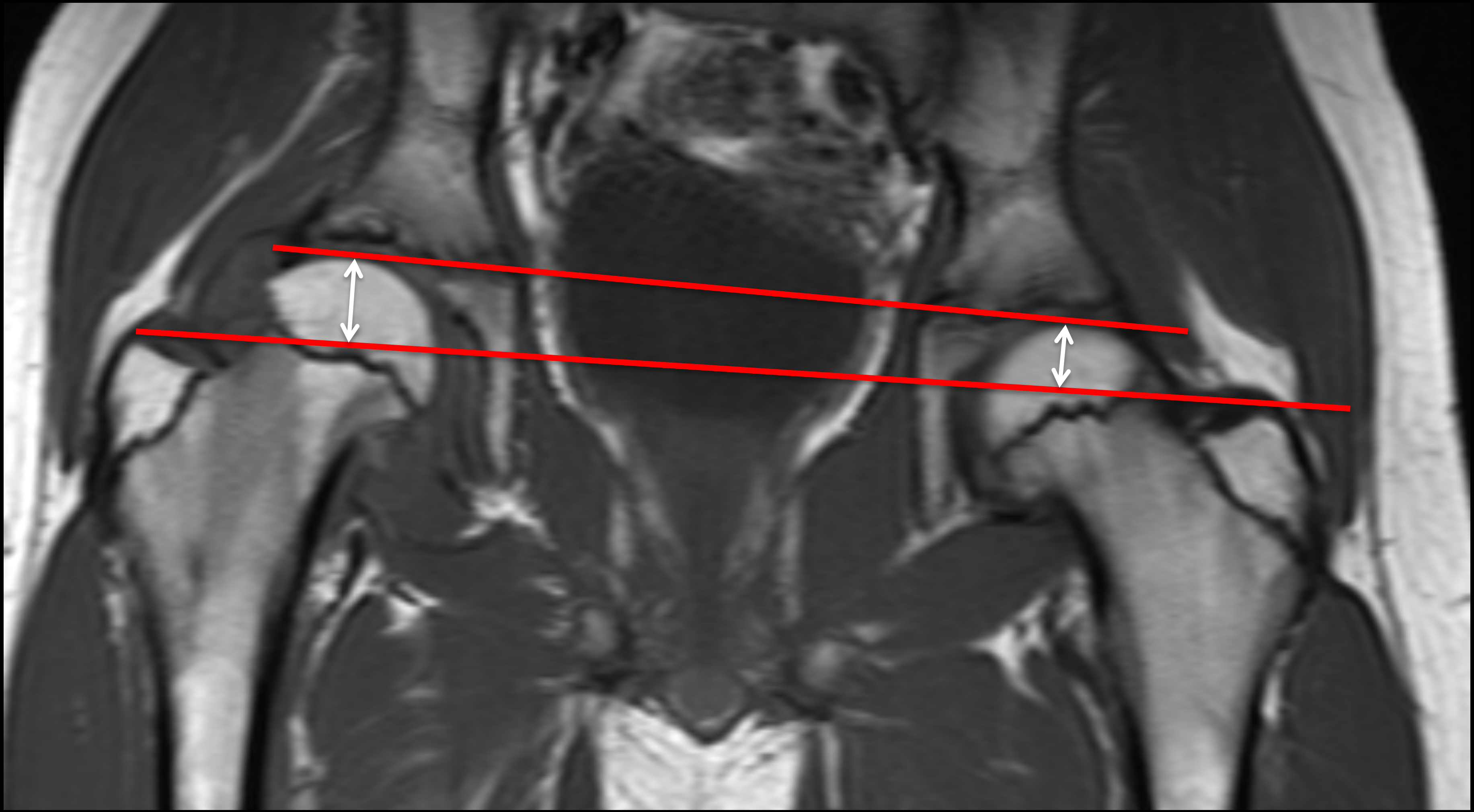

For trochanteric-femoral height, draw two lines, one along superior femoral head and one between the greater trochanters. Measure distance between the two lines at the femoral heads
